# Shut down of the South American summer monsoon during the penultimate glacial

**DOI:** 10.1038/s41598-020-62888-x

**Published:** 2020-04-15

**Authors:** Paula A. Rodríguez-Zorro, Marie-Pierre Ledru, Edouard Bard, Olga Aquino-Alfonso, Adriana Camejo, Anne-Laure Daniau, Charly Favier, Marta Garcia, Thays D. Mineli, Frauke Rostek, Fresia Ricardi-Branco, André Oliveira Sawakuchi, Quentin Simon, Kazuyo Tachikawa, Nicolas Thouveny

**Affiliations:** 1 ISEM, Univ Montpellier, CNRS, EPHE, IRD, 34095 Montpellier, France; 2 CEREGE, Aix Marseille Univ, CNRS, IRD, INRAE, Coll France, 13545 Aix-en-Provence, France; 3 Institute of Geosciences, University of Campinas, 13081-970 Campinas, Brazil; 4 University of Bordeaux, UMR 5805 CNRS EPOC, 33615 Pessac, France; 5 Institute of Geosciences, University of São Paulo, São Paulo, Brazil

**Keywords:** Palaeoecology, Palaeoclimate

## Abstract

We analysed changes in mean annual air temperature (MAAT), vegetation and biomass burning on a long and continuous lake-peat sediment record from the Colônia basin, southeastern Brazil, examining the responses of a wet tropical rainforest over the last 180 ka. Stronger southern atmospheric circulation up to the latitude of Colônia was found for the penultimate glacial with lower temperatures than during the last glacial, while strengthening of the South American summer monsoon (SASM) circulation started during the last interglacial and progressively enhanced a longer wet summer season from 95 ka until the present. Past MAAT variations and fire history were possibly modulated by eccentricity, although with signatures which differ in average and in amplitude between the last 180 ka. Vegetation responses were driven by the interplay between the SASM and southern circulation linked to Antarctic ice volume, inferred by the presence of a cool mixed evergreen forest from 180 to 45 ka progressively replaced by a rainforest. We report cooler temperatures during the marine isotope stage 3 (MIS 3: 57-29 ka) than during the Last Glacial Maximum (LGM: 23–19 ka). Our findings show that tropical forest dynamics display different patterns than mid-latitude during the last 180 ka.

## Introduction

**Figure 1:**
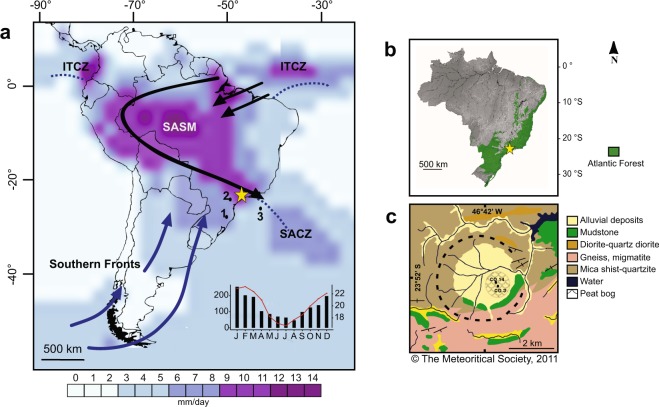
Regional setting and study site. (**a**) Map of South America, showing the distribution of precipitation, plotted as the long-term mean annual precipitation from 1981 to 2010 using the data from the Global Precipitation Climatology Project (GPCP)^85^. Highlighting the South American summer monsoon (SASM; black arrows), and the position of the Intertropical and South Atlantic convergence zones (ITCZ and SACZ; blue dashed lines) during the austral summer in South America (DJF). Blue arrows show the northernmost position of the year-round southern fronts. Mean monthly precipitation at Colônia^29^ (black bars) and mean monthly temperature in São Paulo^30^ (red line) are shown at the bottom of the panel. The numbers refer to the regional datasets to be compared with Colônia (yellow star); (1) Botuverá^9^ (2) Santana^42^ (3) Sea surface temperature record GL-1090^39^. (**b)** Map showing the distribution of the Atlantic forest in Brazil. (**c**) Geology of the Colônia structure showing the location of the sediment cores CO3 and CO14 (adapted from Riccomini *et al*.^24^).

Tropical rainforests (e.g. Amazon, Atlantic forest) cover 49% of the South American territory^1^ but are of global importance as they account for nearly 50% of total carbon storage^2^ and the water cycle^3,4^, and host between half and two-thirds of the world’s species^5^. The Atlantic forest domain offers a unique opportunity to test palaeoclimatic and palaeoenvironmental dynamics in tropical rainforests, because its floristic composition has been determined by fluctuating climatic conditions in an altitudinal gradient^6^ (from 0 to 2,900 m a.s.l.). The forests are located on a long latitudinal gradient along the Atlantic Ocean coast (0° to 23° S) where the intertropical convergence zone (ITCZ) and the SASM control the precipitation regimes (Fig. 1). Recent studies showed that long-term spatial variations of the hydroclimate led to different SASM responses in the Neotropics^7,8^. In Southeastern Brazil, the SASM appears to be paced by a precession signal^9,10^. However, the question whether the tropical rainforests in South America expanded, retreated or changed their floristic composition during glacial periods remains mostly unanswered due to data scarcity and model discrepancy^11–13^. Understanding ecosystem responses to climatic transitions such as those observed during glacial cycles is important when considering the long-term implications of the current climatic crisis (e.g. rapid increase in temperature, sea level and atmospheric CO_2_), because the future of tropical ecosystems indeed depends on how fast they adapt to the different drivers of change over time.

So far, only four lowland rainforest pollen records covering more than one glacial-interglacial cycle exist. In northern Australia and West Africa, the general forest retreat corresponded to a worldwide decrease in mean annual precipitation during glacial periods^14,15^. In northern Amazonia (Lake Pata), the rainforest continued throughout the last glacial-interglacial with no significant difference in floristic composition between marine isotopic stage 6 (MIS 6: 191 to 135 ka) and MIS 5 (135 to 71 ka)^16^. The Southern Hemisphere records (Lynch crater^14^ and Colônia^17^) show that mixed evergreen rainforests expanded and were more continuous during the penultimate glacial period (MIS 6) than during the last glacial period (MIS 4, 3, 2: 71 to 14 ka). Contrarily, northern mid-latitude records show forest expansion phases occurring only during interglacials^18^. It has been suggested that the Antarctic ice volume and atmospheric _p_CO_2_ modulate the expression of glacial periods in the Southern Hemisphere, particularly the dynamics of the subtropical front and ITCZ^19,20^. This highly dynamic system supplies moisture for tropical precipitation in a round-trip circuit driven by obliquity cycles^21,22^. However, if obliquity drives Southern Hemisphere climate and Antarctic sea ice expansion^23^, the tropical precipitation cycle appears to be mainly driven by precession^9^. One can therefore wonder how the southern rainforest responded to differences in the expression of glacial severity between palaeoclimatic forcings such as temperature and hydroclimate.

Here, we explore the responses of a wet tropical forest in the Atlantic forest domain to changes in MAAT and precipitation during the last 180 ka. We compare the observed regional responses with Southern Hemisphere climate records (Antarctic ice volume and _p_CO_2_) to characterise their respective behaviours.

## Study area

The Colônia basin (23°52′03″S and 46°42′27″W, ca. 700 m a.s.l.) is a depression surrounded by an annular rim with hills reaching 125 m (ca. 900 m a.s.l) (Fig. 1). The crater-like structure was formed in crystalline basement rocks, mostly granitic gneiss, some schist and quartzite of Neoproterozoic age (600–700 Ma), presumably by a meteorite impact^24,25^. The inner structure is filled by alluvial sediments capped by a swamp partially drained by the Vargem Grande stream, which flows eastward through the eastern rim^24^ (Fig. 1).

The regional climate is strongly influenced by northward shifts of polar air masses that result in permanent drizzle and moisture in winter^26,27^ (JJA), while during austral summer (DJF) it is affected by the SASM and the position of the South Atlantic Convergence Zone (SACZ)^28^ (Fig. 1). Mean annual precipitation is 1,600 mm, including two months with less rainfall in austral winter (a short dry season in July and August), although natural fires do not occur during this period due to high moisture availability in the area. The mean annual temperature is 20 °C and the average winter temperature is ~17 °C (Fig. 1)^29,30^. The long-term (from 1981 to 2010) mean monthly temperature anomalies between the city of São Paulo^30^ (where Colônia basin is located) and sea surface temperature (SST) near the coast^31^, reflect less negative differences (−0.2 ± 0.6) during the peak of the influence of the SASM (DJF). The anomalies become more negative (−6 ± 0.5) during winter (JJA) when the precipitation is lower in the region (weaker SASM).

The rim surrounding the Colônia basin is covered by Atlantic rainforest mainly represented by the families Myrtaceae, Rubiaceae, Bromeliaceae, Podocarpaceae, Arecaceae, along with other evergreen forest species. The swamp located in the centre of the basin is dominated by Poaceae, Xyridaceae (*Xyris)*, Lentibulariaceae, Sphagnaceae (*Sphagnum)* and Asteraceae^32^.

## Results

In 2014, a 14-meter sediment record (CO14) was recovered in the swampy area of Colônia using a D-section Russian corer^25^. A sharp sedimentological change at 850 cm revealed a transition from a lacustrine to a peat environment (Supplementary Fig. S1). Distinct sedimentary deposit regimes were characterised and differentiated using X-Ray fluorescence measurements (XRF) (Supplementary Fig. S9). Biological proxies including pollen, branched glycerol dialkyl glycerol tetraethers (brGDGTs) and microcharcoal content were used to reconstruct the vegetation, MAAT and biomass burning. The Colônia composite pollen record contains data from two parallel sections (CO3 and CO14). CO3^17^ was used to compare new proxies of CO14 from mid-MIS 5 until present (see Methods and Supplementary Figs. S5 and S6). Pollen interpretations are based on the arboreal and non-arboreal pollen curve, cool and moist taxa such as *Araucaria* and *Podocarpus*, the most relevant evergreen forest taxa indicators (Anacardiaceae, *Ilex*, *Myrsine*, Myrtaceae) and semi-deciduous taxa indicators (*Alchornea, Celtis*, Euphorbiaceae, Malpighiaceae and Melastomataceae)^33,34^. MAAT reconstruction is based on the brGDGTs temperature calibration from Naafs *et al*.^35^, with temperature values ranging from 13.5 to 26 °C (see Methods section and Supplementary Fig. S7 for a discussion on the GDGTs sources and calibration at Colônia). Biomass burning interpretations are based on microcharcoal concentration data expressed as the total number of microcharcoal particles per gram of dry sediment (nb.g^−1^) (see Methods section). The chronological framework was obtained using a Bayesian approach^36^ (See Methods section and Supplementary Fig. S1). It relies on 22 radiocarbon dates (Supplementary Table S1), the Laschamp^37^ geomagnetic excursion and one optically stimulated luminescence (OSL) date as a validation point for the chronology (Supplementary Table S2). Additionally, the obtained MAAT was fitted to the benthic δ^18^O LR04 stack^38^ using three changes in temperature as tie points (See Methods section, Supplementary Fig. S2, Table S3). The obtained chronology spans the last 180 ka, with a very well defined age-depth model at the top of the core (approximate mean uncertainty of ±1.1 ka) and a loose model below the ^14^C limit (approximate mean uncertainty of ±12 ka) (Supplementary Fig. S1).

**Figure 2:**
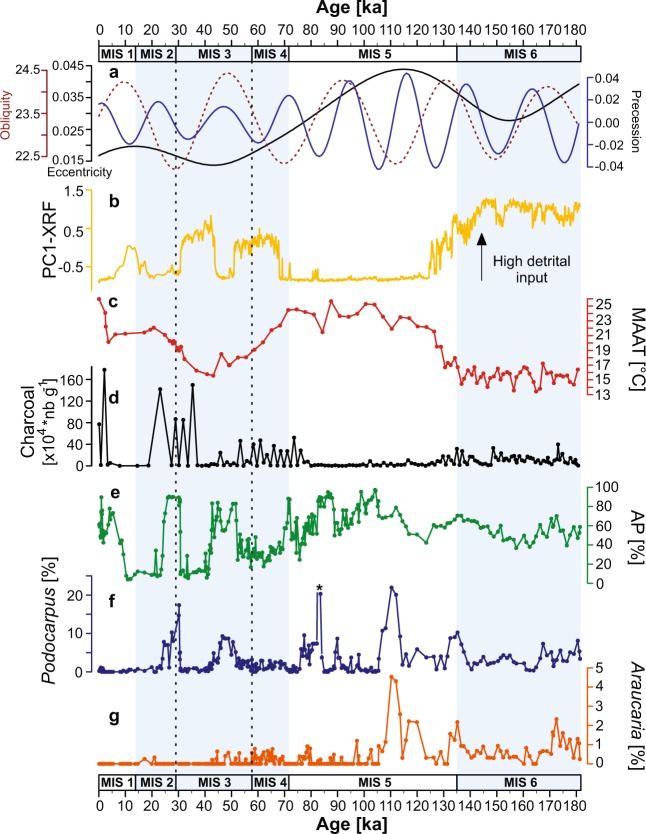
Summary of palaeoecological proxies from Colônia. (**a**) Orbital parameters for latitude 23°52′03″S using La2004^86^. (**b**) PC1 of the intensity of the XRF elements from CO14. (**c**) Reconstructed MAAT (CO14). (**d**) Biomass burning from CO14. (**e**) Arboreal (AP) pollen percentages from CO3 and CO14. (**f**) *Podocarpus* pollen percentages from CO3 and CO14; *reflects a peak of ca. 60% (see Methods section). (**g**) *Araucaria* pollen percentages from CO3 and CO14. The marine isotopic stage boundaries ages are based on the LR04 δ^18^O stack^38,87^. The boundary between MIS 5 and 6 is based on Henderson and Slowey^88^.

**Figure 3:**
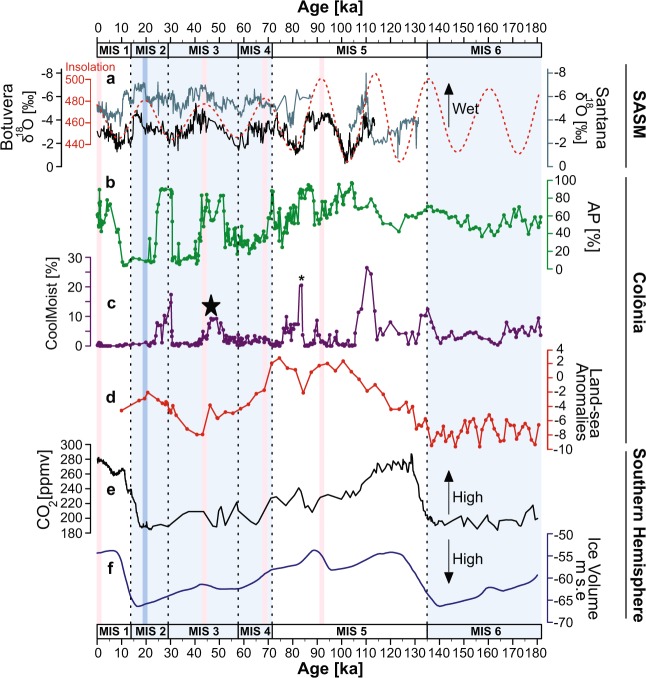
Regional comparison during the last 180 ka. (**a**) Speleothem records (Botuverá (black) and Santana (gray))^9,42^ plotted with insolation (W/m^2^) at latitude 23°52′03″S using La2004^86^ (red dashed line). (**b)** Arboreal pollen percentages (AP) from Colônia (CO3 and CO14). (**c**) Percentage curve of the sum of cool moist conifers *Araucaria* and *Podocarpus*; the black star locates the last continuous occurrence of *Araucaria*, and asterisk represents ca. 60% of *Podocarpus* (see Methods section). (**d**) Land-sea temperature gradient. (**e**) *p*CO_2_ (Lüthi *et al*.^89^). (**f**) Total ice volume represented in m s.e. (meters sea-level equivalent) for the Antarctic ice sheet^45^. The marine isotopic stage boundaries ages are based on the LR04 δ^18^O stack^38,87^. The boundary between MIS 5 and 6 is based on Henderson and Slowey^88^. Pink bands highlight the maximum insolation peaks of influence of the SASM system during the last 95 ka. The blue band also highlights the maximum insolation peak but emphasises the differences in Colônia’s proxies compared with the regional datasets.

Changes from glacial to interglacial are reflected in the temperature brGDGTs-derived curve (MAAT), biomass burning and the first principal component (PC1) calculated using the XRF core scanning dataset (PC1 accounts for 79% of total variance) (Fig. 2 and Supplementary Fig. S9). Land-sea temperature gradient between the anomalies from the MAAT and the SST GL-1090 record^39^ (See Methods section and Supplementary Fig. S8) reflects sustained changes between MIS 5, MIS 2-3-4 and MIS 6. Negative numbers imply relative cooling on land and thus less moisture transport in the region (Fig. 3).

The penultimate glacial (MIS 6) was characterised by the coolest temperatures with an average MAAT of 15.6 ± 1 °C and full expansion of the cool mixed evergreen forest (40–70%). The forest was dominated by *Myrsine*, Myrtaceae, *Araucaria* and *Podocarpus*, and semi-deciduous taxa including Melastomataceae, Malpighiaceae and *Celtis* (Fig. 2 and Supplementary Fig. S5). Open vegetation (30–60%) was dominated mainly by Poaceae and Asteraceae. During MIS 6, biomass burning was on average 11.5 ± 8 × 10^4^ nb.g^−1^. In comparison, the last glacial period (MIS 4, 3, 2) was warmer with an average temperature of 19.5 ± 2.2 °C and was characterised by gradual temperature changes. During that period, MAAT progressively decreased from 24 °C to a minimum temperature of 15.6 °C at 43 ka, and then increased again up to 22 °C from 41 to 21 ka, meaning temperatures were lower during the MIS 3 than during the LGM^40^. Two fire episodes occurred during the last glacial period with contrasted biomass burning from ca. 71 to 50 ka (16 ± 17.7 × 10^4^ nb.g^−1^ on average) and from ca. 35 to 23 ka (66.5 ± 66.3 × 10^4^ nb.g^−1^ on average). Additionally, the vegetation was characterised by fluctuations from mixed evergreen forest to open areas dominated by grasslands^17^. During MIS 4 (71–57 ka) the landscape was mainly characterised by grasslands (9–66%) although a mixed evergreen forest persisted. MIS 3 (57–29 ka) was characterised by early expansion of grasslands and an increase in mixed evergreen forest between 50 and 40 ka, followed by a decline in forest until 30 ka. During MIS 2 (29–14 ka), the mixed evergreen forest reached full expansion between 30 and 25 ka, and grasslands were dominant during the late glacial. Variations in detrital inputs detected by the PC1 from the XRF core scanning (e.g. Si,Ti, inc/coh) suggest increased erosion rates during the dry periods reported by speleothem records and forest contractions over the last glacial period (see description of the sediment in Supplementary Material).

## Discussion

### Palaeoclimate and vegetation responses to glacial-interglacial cycles

During the penultimate glacial, cooler temperatures in Colônia (on average 4 °C lower than during the last glacial) favoured the presence of permanent cool mixed evergreen forest with conifers *Araucaria* and *Podocarpus* associated with strong southern circulation towards the tropics. This has been assumed since recent estimates based on modern pollen climate calibration in the Atlantic forest domain showed that the continuous strong northward shifts of southern air masses were linked to expansion of the cool mixed forest^27,34,41^. In addition, a contrasting land-sea anomalies gradient (−7.4 ± 1.2) could explain a reduced SASM intensity to the study site, due to the effect of cooler land temperatures compared to the SST during that period (Fig. 3 and Supplementary Fig. S8). Such dry effect can be observed in the relatively frequent fires, the high frequency of open vegetation and semi-deciduous forest taxa (Supplementary Fig. S5). On the other hand, the presence of the mixed evergreen forest points out that there was still high moisture input (Fig. 2, Supplementary Fig. S5). Today, natural fires do not occur in the area, not even during the dry winter season (July-August), because of the permanent moisture rates (Fig. 1). We therefore suggest that during the MIS 6, SASM circulation was weaker than today, or nearly absent, implying a summer dry season. This hypothesis relies on observations from two nearby speleothem records showing that the strength of the SASM over the last 112 ka is related to insolation in austral summer at precession scale^9^ (Fig. 3).

Temperatures at Colônia were warmer during the MIS 5 (130–71 ka) (increase of 7 °C) than during the previous glacial period (MIS 6) at 23.1 ± 1.8 °C on average. Evidence for the highest continuous expansion of the mixed evergreen forest is the presence of cool moist taxa especially *Araucaria* between 125 and 95 ka (Fig. 2). In this context, although forest expansion would be expected in the high summer insolation phase^17^ (more precipitation as inferred from the speleothem records), the abundance of the cool taxa *Araucaria* and *Podocarpus* suggests that other factors prevailed during the warm last interglacial period (Figs. 2 and 3). This pattern was probably linked to enhanced extra tropical circulation and abundant winter precipitation, as revealed by the Santana speleothem record from 130 to 112 ka^42^. On the other hand, the dominance of austral summer insolation on precipitation from 95 ka as also revealed by the speleothem records^9^, significantly reduced biomass burning (from 95 to 80 ka: 0.9 ± 0.8 × 10^4^ nbg^−1^ on average) and increased tree pollen frequencies. From early to mid- MIS 5, land-sea temperature differences were gradually reduced (−0.8 ± 2.4), which is likely reflecting a stronger SASM influence during summer in the area^43^ (Fig. 3).

The last glacial (MIS 2, 3 and 4) was associated with different global changes in ice volume, temperature and sea level^44,45^. An open landscape associated with drier climate conditions and two phases of erosion (high PC1) characterised the late glacial during low precession phases (with weak SASM activity^9^) and no extra tropical circulation reached Colônia’s latitude. Additionally, the retreat of the cool and moist taxa indicators points to reduced moisture availability in winter. Similarly, the temperature gradient between land and sea increased (−4 ± 2), likely reflecting a decrease on moisture input due to a weak SASM activity. The first phase of biomass burning (71 to 50 ka) suggests that fires were caused by a change in seasonality with an increase in precipitation during austral winter and a decrease during the austral summer. Additionally, the presence of a cool mixed evergreen forest in the region with *Araucaria* and *Podocarpus*^34,41,46^ points to the influence of southern air masses in southeastern Brazil. The strengthening of the SASM and high summer precipitation during a high precession phase^9^ between 50 and 40 ka reduced biomass burning and increased the mixed evergreen forest cover (Fig. 2, Supplementary Fig. S5), also corresponding to bigger differences between land and sea temperatures. The last continuous occurrence of *Araucaria* observed at 45 ka shows the declining influence of the extra tropical circulation and the increasing dominance of the SASM system in southeastern Brazil. The second episode of biomass burning (35 to 23 ka) in this glacial period is the biggest reported in the record. It started with a low precession phase and a weaker SASM (drier conditions), which does not appear to be linked to grassland or forest expansion (30–25 ka) (Fig. 2). Temperatures at Colônia gradually increased from 15.7 to 22 °C between mid-MIS 3 and mid-MIS 2. Neither the Colônia nor the SST records (GL-1090)^39^ contain evidence for cooling during the Last Glacial Maximum, but earlier during the MIS 3.

The relatively cooler Early Holocene, with a slight decrease of 1 °C in MAAT, was followed by a rise in temperature from 3.6 ka on. Renewed biomass burning from 2 ka on, including the highest peak of the record (178 × 10^4^ nb.g^−1^), coincided with forest regression between 2.3 and 1.6 ka.

### Effect of the Southern Hemisphere on the Tropics

The observed environmental and climatic changes between glacial-interglacial periods in Colônia could be orbitally driven at eccentricity scale although a longer record is needed to further test our hypothesis (Fig. 2, Supplementary Fig. S10). Additionally, due to the latitudinal location^47^, rainforest expansion and contraction phases were a unique response as a result of a combination of winter seasonality driven by obliquity along with the SASM driven by precession (Figs. 2 and 3, Supplementary Fig. S10). However, additional mechanisms other than insolation cycles must be inferred to explain changes in fire activity or the continuous presence of a cool mixed forest from the penultimate glacial period (MIS 6) until the mid-MIS 5.

The timing and climatic variations observed in the CO14 record could be linked to the differences between temperature and precipitation observed at different latitudes as suggested by models^48^. We propose that the influence of variations in the Antarctic ice sheet on the tropical hydrological cycle affected the floristic composition of the rainforest from the penultimate glacial to mid-MIS 5 (180 to 95 ka). Based on studies of the last glacial in the Southern Hemisphere^44,49,50^, we propose that the combination of low _P_CO_2_, extended Antarctic ice volume, and an equatorward shift of the westerlies prevented the tropical circulation from influencing the Colônia site from MIS 6 to mid-MIS 5^49^. Those conditions would have allowed the development of a mixed cool evergreen and semi-deciduous forest with no modern analogue^34^, along with fire activity during a drier summer season (Fig. 3, Supplementary Fig. S5).

At the beginning of MIS 5, a poleward shift of the westerly belt, an increase on _p_CO_2_ and upwelling in the south Atlantic enhanced tropical circulation during austral summer^44,50^, although not enough to remove winter precipitation inputs^27,51–53^ in the Colônia area (inferred by the presence of *Araucaria* and *Podocarpus*). Thus, the Antarctic ice sheet configuration, which controls the position of the subtropical front^19^, would explain the shift from dominance of extra tropical circulation^27^ during 180 to 95 ka to dominance of the SASM system on the climate patterns from 95 ka onwards (Fig. 3). Throughout MIS 5, during Antarctic ice sheet contractions, the intensity and amplitude of winter rainfall also progressively decreased and a transition phase (80 to 60 ka) characterised by broad changes in forest composition and reactivation of biomass burning, is explained by oscillations in the interplay of the southern air masses and the SASM system.

During the last glacial, the retreat of the cool mixed evergreen forest coincided with a gradual increase in temperatures attributed to the increasing influence of the tropical circulation paced by the precession signal (Fig. 3), while the progressive rise in temperature over the 41 to 21 ka interval was antiphased with low _*P*_CO_2_ values and high Antarctica ice volume. Our research highlights the teleconnections between low and high latitude regions and the growing influence of the SASM circulation on climate and vegetation in the southern tropics. The changes in seasonal rainfall distribution from winter centred with continuous fire activity and low temperatures during the penultimate glacial, to summer centred rainfall distribution during the last glacial with major fluctuations in fire activity and temperature, shaped the composition and distribution of the current tropical forest. Further analyses of a deeper core from Colônia are expected to test the long-term eccentricity pacing on regional environmental and climatic changes, the SASM behaviour during previous glacial stages, and characterise how Antarctic ice volume influenced tropical climate and forest diversity.

## Methods

### Magnetostratigraphy

The half core sections were sampled using u-channels (2 × 2 × 100 cm) for magnetic measurements. Natural, anhysteretic and isothermal remanent magnetisation (NRM, ARM and IRM) were measured at 2-cm depth resolution using a three-axis DC SQUIDS cryogenic magnetometer (2 G 760 SRM) installed in a magnetically shielded room at CEREGE. Alternating field (AF) demagnetisation was applied stepwise at 5, 10, 15, 20, 30, 40, 50, 60 and 80 mT. ARM was imparted in an 80 mT AF and a 0.1 mT direct field (DF) and demagnetised at the same steps as the NRM. IRM was imparted by passing u-channels though a 0.6 T Halbach cylinder to obtain a saturation IRM for magnetite grains (SIRM), and then demagnetised stepwise from 5 to 120 mT. NRM, ARM and IRM display strong stability during AF treatment likely associated with the presence of high coercivity minerals and the small magnetic grain size fraction (single- to pseudo single-domain Ti-magnetite). The demagnetising curves reveal stable intensity, with medium destructive field (MDF) of the NRM barely determined, and stable direction components. Data from the ends of the core (from two to four measurement points) were cleaned to reduce the edge effect associated with the pickup coils response function. An average inclination shallowing of 24° compared to the geocentric axial dipole (GAD) configuration (−12° instead of −36°) likely results from compaction of the organic rich sediments. NRM intensity measured at the 30 mT AF demagnetisation step was divided by ARM intensity to obtain a relative palaeointensity index (RPI). The largest inclination deviation, which reached −87° at a depth of 1.72 m, corresponds to low RPI values and was attributed to the Laschamp^37^ excursion, which also fits independent radiocarbon dating. Two other significant inclination deviations associated with low RPI are observed at 3.5 and 6.7 m, respectively.

### Radiocarbon dating

A total of 22 samples of bulk peat sediment were analysed at Artemis LMC14 (Gif-sur-Yvette, France) (Supplementary Table S1). The samples were examined under binoculars to exclude possible macro contaminants and were pre-treated chemically using HCl (0.5 N) and NaOH (0.1 N) at 80 °C. Reported ^14^C activity is expressed as pMC (percent modern carbon), normalised to δ^13^C of −25‰. The reported ages were calibrated using the IntCal13 curve^54^. The radiocarbon age of sample SacA41593 was calibrated using a post-bomb curve for the Southern Hemisphere^55^.

Calibrations and age-depth modelling were performed using R^56^ and the RBacon package^36^.

### Luminescence dating

Luminescence dating was performed at the Luminescence and Gamma Spectrometry Laboratory (LEGaL – *Instituto de Geociências, Universidade de São Paulo*, Brazil) (Supplementary Table S2). Using opaque syringes, two samples were collected from sandy intervals located at depths of 1 m and 11 m. The surface of the sample that had been exposed to light (ca. 1 cm) was removed under subdued amber light conditions and a total of 20.3 cm^3^ sediment volume per sample was used for luminescence dating. Grains of quartz and K-feldspar in the 180–250 μm grain size fraction were isolated using standard procedures^57^. Multigrain aliquots of quartz and K-feldspar were measured in a Risø TL/OSL DA-20 reader equipped with blue and infrared (IR) LEDs for light stimulation and a ^90^Sr/^90^Y beta radiation source (0.084 Gy/s dose rate). First, equivalent doses were estimated using the optically stimulated luminescence (OSL) signal of quartz^58^ detected in the ultraviolet band. The natural OSL signal of the quartz aliquots taken from sample L0247 (1 m depth) was below saturation, but sample L0250 showed saturated natural OSL signals. Consequently, the sediment burial ages of L0250 were determined using the 310 °C isothermal thermoluminescence (ITL) signal of quartz^59^ and post-infrared stimulated luminescence (pIRIR) at 290 °C (Buylaert *et al*.^60^) of K-feldspar (detection in the blue light window). Sample L0250 also showed a saturated natural pIRIR signal, with minimum equivalent dose (2D_0_) of ~408 Gy. The pIRIR signal consequently only gave a minimum age for sample L0250, and its burial age was determined using the ITL signal from quartz, which was below saturation. We calculated a minimum age based on the IRSL at 50 °C signal (IR50) measured in the first step of the pIRIR at 290 °C dating protocol to constrain the quartz ITL age. The IR50 age is given without correction for athermal fading. The performance of all luminescence dating protocols used in this study was checked through dose recovery tests applied to quartz and K-feldspar aliquots bleached under a solar simulator lamp. The dose recovery test for the quartz OSL signal was performed using quartz aliquots from an adjacent core because sample L0247 did not have sufficient quartz in the target grain-size fraction. The quartz from sample L0247 was kept to estimate equivalent doses. The calculated-to-given dose ratio for the quartz OSL signal was obtained using a preheating temperature of 200 °C and the given dose of 150 Gy was 0.99 ± 0.07 (four aliquots). Given-to-calculated dose ratios for the pIRIR at 290 °C and ITL signals were respectively 1.03 and 1.23 for a given dose of 300 Gy. Equivalent doses of each sample were calculated according to the Central Age Model^61^. Equivalent doses for the sample were calculated with aliquots following acceptance criteria to check corrections for sensitivity changes (recycling ratio within 1.0 ± 0.1) and thermal transfer (recuperation less than 5%). We underline the fact that equivalent doses were calculated using four to six aliquots because of the reduced volume of the available sample.

To estimate the radiation dose rate, natural radionuclide (^238^U, ^232^Th and ^40^K) concentrations were measured by high-resolution gamma ray spectrometry in a high purity germanium detector (HPGe, relative efficiency of 55% and energy resolution of 2.1. KeV) mounted on an ultra-low background shield (Canberra Instruments). Samples (L0250:11 g; L0247:17.4 g) were measured after storage for at least 21 days in sealed plastic containers for radon equilibrium. U, Th and K concentrations were converted into dose rates^62^. The contribution of cosmic radiation to the dose rate was calculated using conversion factors based on the latitude, longitude, altitude, and depth of the samples^63^.

The ITL L0250 date was not used in the model and chronological framework due to its large uncertainty (Supplementary Table S2).

### Colônia chronology

To check the overall consistency of the Colônia chronological framework, we used three independent posterior validation points:

1. The OSL age of 31.4 ± 4.3 ka agrees with radiocarbon dating and fits the Bayesian envelope;
2. we fine-tuned the RPI curve to two independent palaeointensity reference curves^64,65^ assuming that the three inclination deviation/low RPI levels record the Laschamp^37^ (41 ka) (supported by the radiocarbon dated section in CO14), Norwegian-Greenland Sea^66^ (64 ka) and post-Blake geomagnetic excursions^67^ (100 ka). Despite legitimate doubts concerning the RPI signal due to complex magnetic mineralogy which prevented its use for straightforward reliable RPI wiggle-matching, the visual relationship within the peat section is acceptable (0–9 m; Supplementary Fig. S3) and support the age model we obtained, i.e., 100 ka at 6.7 m according to the RPI matching versus 109 ± 11 ka derived from the independent chronology;
3. the reconstructed MAAT curve shows that the sediments from CO14 are not older than the MIS 7 (191 ka) (Supplementary Fig. S2), deduced by the absence of a contrasting temperature change at the bottom part of the record.

After this validation, we also fitted the MAAT curve to the benthic δ^18^O LR04 stack^38^ using three changes in temperature as tie points (Supplementary Fig. S2, Table S3).The main objective was to refine our chronology for the part beyond the radiocarbon dated section. The selected tie points correspond to the transition from MIS 6 to 5, the onset of the MIS 5e, and the transition from MIS 5/4. Each tie point contains an arbitrary uncertainty of ± 5 ka, acknowledging the differences between both curves.

The age-depth model from the CO3 record was re-calculated based on the five published radiocarbon dates using a Bayesian approach (Supplementary Fig. S4, Table S4) (RBacon^36^). To be able to compare the new proxies and the pollen section from CO14, the CO3 record was aligned to CO14 based on both independent ^14^C age-depth models (CO3 and CO14). The section with no radiometric control (182 to 752 cm) in the record CO3 was aligned to the CO14 record assuming that both records could have the same sedimentation rate, as they are located close one to another (5 m). Although the two records could hypothetically have similar sedimentation rates due to their proximity, and as shown by the radiocarbon dated section, we acknowledge higher uncertainty at the bottom part of both records, particularly within the CO3 record. For palynological purposes, the two records were merged based on the similarities of the pollen percentages in each data set (CO3 and CO14) (See section on pollen analyses and Supplementary Fig. S6).

### XRF analyses

X-Ray Fluorescence (XRF) scans were performed to determine the variability of Al, Si, S, K, Ca, Ti, Fe, Rb and Zr using an ITRAX scanner (Cox Analytical Systems) at CEREGE (France). To optimise the count response of light and heavy elements, scans were performed using a Cr tube set at 30 kV and 40 mA, and a Mo tube at 30 kV and 45 mA, respectively. The spatial resolution of XRF measurements was 1 mm with a counting time of 15 seconds. The ratio of incoherent to coherent responses (inc/coh) obtained with the Mo tube corresponds to the Compton to Rayleigh scattering ratio and represents the relative abundance of light and heavy elements^68,69^. Transmitted X-ray images were obtained using the Mo tube set at 40 kV, 35 mA, the counting time was 500 ms for a resolution of 200 μm.

### brGDGTs temperature curve

Mean annual air temperature (MAAT) is based on the analysis of branched glycerol dialkyl glycerol tetraethers (brGDGTs) following the method described by Hopmans *et al*.^70^ and adapted at CEREGE by Davtian *et al*.^71,72^. A total of 92 sub-samples from CO14, each weighing between 0.1 to 0.9 grams were freeze-dried, ground and extracted using an accelerated solvent extraction system (ASE350 Dionex-Thermo Fisher) at 120 °C, 100 bars and a mixture of dichloromethane and methanol (9 :1, v/v). Extracts were then separated into apolar and polar fractions following the method described by Sanchi *et al*.^73^. The polar fraction with the brGDGTs was analysed by high-performance liquid chromatography-atmospheric pressure chemical ionisation-mass spectrometry (HPLC-APCI-MS; Agilent 1250 Infinity HPLC coupled with an Agilent 6120 quadrupole mass spectrometer).

We applied the global peat-specific temperature calibration protocol developed by Naafs *et al*^35^. (MAAT°C = −23.05 + 52.18*MBT′5ME), which led to MAAT values of about 24 °C for samples from the top of the core. This value is higher than the modern MAAT (20 °C), with a seasonal range between 15 and 25 °C at Colônia (Supplementary Fig. S7). However, the brGDGTs-based temperature is still compatible with the modern MAAT, considering the calibration uncertainty (root mean square error (RMSE) ≈ 4.7 °C). The brGDGTs-based temperature values correspond to the summer temperature which is also the wettest period of the year (Fig. 1); both parameters being favourable to the brGDGT producers. Using a recent brGDGTs soil calibration^74^ (MAAT°C = −8.57 + 31.45*MBT′5ME; RMSE ≈ 4.8) results in very similar MAAT estimates for the glacial period. By contrast, the application of a calibration for aquatic production of brGDGTs in lakes (e.g. Russell *et al*.^75^; MAAT = −1.21 + 32.42*MBT′5ME; RMSE ≈ 2.44) led to very high palaeotemperatures values ranging between 27 and 29 °C during MIS 5 and 29 °C for the core top, much higher than the modern MAAT and seasonal range.

In order to estimate the origin of tetraethers at Colônia and selection of the most appropriate calibration curve, we used the ratio of specific GDGTs and brGDGTs as qualitative indicators of terrestrial organic matter versus aquatic production (Supplementary Fig. 7). First, we applied the Branched and Isoprenoid Tetraether (BIT) index, which varies between 0 and 1 (1 being an entirely terrestrial origin)^71,76–78^ and the brGDGTs ratio ∑IIIa/∑IIa (∑IIIa = sum of isomers of the ion trace [M + H] + 1050 and ∑IIa = sum of isomers of the ion trace [M + H] + 1036) with values <0.5 considered as terrestrial and >1.2 considered of aquatic origin^79,80^. For both, the lacustrine and peat sections, high BIT index values (0.95 to 1) and very low ∑IIIa/∑IIa ratios (0.04 to 0.22) are the signature of a terrestrial origin for brGDGTs in the Colônia record. In the peat section, brGDGTs production is thus considered as *in-situ*^35,77^, whereas in the lake section accumulated during humid glacial periods, the brGDGTs probably came from the erosion of soils on the borders of the lake or its catchment. This origin is supported by increased PC1 of XRF elemental data indicating siliciclastic sediments deposition during the lacustrine phase (Fig. 2).

Consequently, we applied the peat calibration of Naafs *et al*.^35^ to the entire CO14 core. The calibration leads to cooling of about 8 °C for the MIS 3, MIS 2 and MIS 6. This glacial cooling is somewhat larger than previous estimations based on noble gases in aquifers, pollen in sediments and mountain snow lines in South America for the LGM (ca. 5 °C)^81^. This difference in amplitude may be linked to the uncertainty of the brGDGTs calibration from Naafs *et al*.^35^.(RMSE ≈ 4.7 °C). Using the lake sediment calibration by Russell *et al*.^75^ would lead to lower glacial cooling of about 5–6 °C.

In any case, the overall shape and relative variation of the brGDGTs palaeotemperature curves are similar using different calibrations for peat, soils and lakes^35,74,75^. This implies that the identification of MIS 1, MIS 2, MIS 5 and MIS 6 is reliable, as confirmed by the correlation with changes in other stratigraphic proxies (e.g. XRF elements intensity, charcoal and pollen data). A similar terrestrial origin of the brGDGTs in both, the peat and lacustrine sediments is demonstrated by high values of the BIT index and low values of the ∑IIIa/∑IIa ratio, which justifies the application of the same calibration throughout the whole core, preferably the global peat calibration as it leads to recent temperatures compatible with that of the present day.

### Land-sea anomalies

The variations of the land-sea temperature gradient have been calculated by subtracting the temperature anomalies with respect to the core top, for the continental MAAT record (based on brGDGTs), and for the marine SST record (based on Mg/Ca in the core GL-1090)^39^. This index is a semi-quantitative approximation which precision depends on uncertainties of the calibrations used for both proxies (±1.2 and 4.7 °C, respectively) and consequently, only systematic and sustained changes are meaningful and to be considered in our study (e.g. contrasts between MIS 5, MIS 2, 3, 4 and MIS 6) (Supplementary Fig. S8).

### Pollen analyses

Pollen analyses were based on two parallel cores separated by a distance of 5 m, CO3^17^ and CO14. Based on the previously described core alignment, and acknowledging the uncertainties from the bottom part of the record CO3, we considered the matching point to be 105 ka (CO3:726 cm and CO14: 690 cm) based on arboreal pollen, Poaceae and *Araucaria* percentage matching curves. We have excluded only 6 samples in the CO3 data set for graphical purposes (Supplementary Fig. S6). Consequently, pollen samples from the interval 180 to 105 ka belong to the CO14 record and pollen samples from 105 ka towards the present belong to the CO3 record^17^.

A total of 57 subsamples were analysed for the CO14 record. We applied an uneven subsampling approach based on knowledge of eight test samples (1263, 1277, 1293, 1299, 1301, 1313, 1327 and 1337 cm depth) of 1 cm^3^ in volume. The samples were collected from the clayey section of the record with high pollen content; the remaining unanalysed section was subsampled using a smaller volume, 0.5 cm^3^. Each sample was treated using standard methods for pollen analysis^82^. Pollen was counted to a minimum of 300 terrestrial pollen types.

A percentage peak of *Podocarpus* from the record CO3 centred at 83 ka (lasted ca.1 ka) is associated to specific climate features with a sharp decrease in MAAT, land-sea gradient and an increase in _p_CO_2_. Pollen assemblages are composed of 9–16% of *Ilex*, 2–8% of Asteraceae and 2–9% of Myrtaceae, and do not relate to any modern analogue of the Atlantic forest today^34^. This suggests that *Podocarpus* expanded locally on the lake shore and therefore its peak is only highlighted with an asterisk in Figs. 2 and 3.

All illustrations and calculations were done using the Psimpoll program^83^ and R^56^.

### Charcoal analyses

A total of 147 subsamples were sampled for microcharcoal analyses according to the methodology of Daniau *et al*.^84^ using a series of chemical laboratory treatments combining hydrochloric acid (HCl), nitric acid (HNO_3_), hydrogen peroxide (H_2_O_2_) and hydrofluoric acid (HF) to remove calcium carbonates, organic matter and siliceous material. The peat section (850 to 5 cm) was treated differently due to the very high concentration of organic remains. A total of 0.5 g of dry peat was sieved with water. Next, 0.2 g of dry sediment (<150 µm) per sample was chemically treated, excluding the step using HF. Microcharcoal particles were counted manually at x 500 magnification.

## Supplementary information

Supplementary Material.

## Data Availability

The CO3 pollen record is available on the NEOTOMA database. The new combined pollen dataset, XRF, MAAT and charcoal data are deposited on the PANGAEA open access data base 10.1594/PANGAEA.907731.

## References

[1] Lewis SL (2006). Tropical forests and the changing earth system. Philos. Trans. R. Soc. B Biol. Sci..

[2] Saatchi SS (2011). Benchmark map of forest carbon stocks in tropical regions across three continents. Proc. Natl. Acad. Sci..

[3] Malhi Y, Meir P, Brown S (2002). Forests, carbon and global climate. Philos. Trans. R. Soc. Lond. Ser. Math. Phys. Eng. Sci..

[4] Marengo JA (2006). On the hydrological cycle of the Amazon basin: a historical review and current State-of-the-art. Rev. bras. meteorol..

[5] Levine NM (2016). Ecosystem heterogeneity determines the ecological resilience of the Amazon to climate change. Proc. Natl. Acad. Sci..

[6] Duarte LDS, Bergamin RS, Marcilio-Silva V, Seger GDDS, Marques MCM (2014). Phylobetadiversity among forest types in the Brazilian Atlantic forest complex. PLoS ONE.

[7] Cheng H (2013). Climate change patterns in Amazonia and biodiversity. Nat. Commun..

[8] Stríkis NM (2018). South American monsoon response to iceberg discharge in the North Atlantic. Proc. Natl. Acad. Sci..

[9] Cruz FW (2005). Insolation-driven changes in atmospheric circulation over the past 116,000 years in subtropical Brazil. Nature.

[10] Deininger M, Ward BM, Novello VF, Cruz FW (2019). Late Quaternary variations in the South American monsoon system as inferred by speleothems—New perspectives using the SISAL database.. Quaternary.

[11] Bennett K, Bhagwat S, Willis K (2012). Neotropical refugia. The Holocene.

[12] Baker PA, Fritz SC (2015). Nature and causes of Quaternary climate variation of tropical South America. Quat. Sci. Rev..

[13] Flantua SGA (2015). Updated site compilation of the Latin American Pollen Database. Rev. Palaeobot. Palynol..

[14] Kershaw AP, Bretherton SC, van der Kaars S (2007). A complete pollen record of the last 230 ka from Lynch’s Crater, north-eastern Australia. Palaeogeogr. Palaeoclimatol. Palaeoecol..

[15] Miller CS, Gosling WD, Kemp DB, Coe AL, Gilmour I (2016). Drivers of ecosystem and climate change in tropical West Africa over the past ∼540,000 years. J. Quat. Sci..

[16] D’Apolito C, Absy ML, Latrubesse EM (2017). The movement of pre-adapted cool taxa in north-central Amazonia during the last glacial. Quat. Sci. Rev..

[17] Ledru M-P, Mourguiart P, Riccomini C (2009). Related changes in biodiversity, insolation and climate in the Atlantic rainforest since the last interglacial. Palaeogeogr. Palaeoclimatol. Palaeoecol..

[18] Litt T, Pickarski N, Heumann G, Stockhecke M, Tzedakis PC (2014). A 600,000 year long continental pollen record from Lake Van, eastern Anatolia (Turkey). Quat. Sci. Rev..

[19] Bard E, Rickaby REM (2009). Migration of the subtropical front as a modulator of glacial climate. Nature.

[20] Khodri, M., Kageyama, M. & Roche, D. M. Sensitivity of South American tropical climate to Last Glacial Maximum boundary conditions: Focus on teleconnections with tropics and extratropics In *Past Climate Variability in South America and Surrounding Regions* (eds. Vimeux, F., Sylvestre, F. & Khodri, M.).**14**, 213–238 (Springer Netherlands, 2009).

[21] Vimeux F, Masson V, Jouzel J, Stievenard M, Petit JR (1999). Glacial–interglacial changes in ocean surface conditions in the Southern Hemisphere. Nature.

[22] Masson-Delmotte V (2010). EPICA dome C record of glacial and interglacial intensities. Quat. Sci. Rev..

[23] Fogwill CJ, Turney CSM, Hutchinson DK, Taschetto AS, England MH (2015). Obliquity control on southern hemisphere climate during the last glacial. Sci. Rep..

[24] Riccomini C (2011). The Colônia structure, São Paulo, Brazil. Meteorit. Planet. Sci..

[25] Ledru M-P (2015). Why deep drilling in the Colônia Basin (Brazil)?. Sci. Drill..

[26] Grimm A, Ferraz S, Gomes J (1998). Precipitation anomalies in Southern Brazil associated with El Niño and La Niña events. Am. Meteorol. Soc..

[27] Garreaud RD (2000). Cold air incursions over subtropical South America: Mean structure and dynamics. Mon Weather Rev..

[28] Garreaud RD, Vuille M, Compagnucci R, Marengo J (2009). Present-day South American climate. Palaeogeogr. Palaeoclimatol. Palaeoecol..

[29] DAEE. *Portal do departamento de águas e energia elétrica*. http://www.hidrologia.daee.sp.gov.br/ (2019)

[30] INMET. *Instituto nacional de meteorologia*. http://www.inmet.gov.br (2019)

[31] Locarnini, R. A. *et al*. *World ocean atlas 2018*. (ed. Mishonov, A.) **1**, 52 (NOAA Atlas NESDIS, 2019).

[32] Garcia RJF, Pirani JR (2005). Análise florística, ecológica e fitogeográfica do núcleo Curucutu, parque estadual da Serra do Mar (São Paulo, SP), com ênfase nos campos junto à crista da Serra do Mar. Hoehnea.

[33] Ledru M-P, Montade V, Blanchard G, Hély C (2016). Long-term spatial changes in the distribution of the Brazilian Atlantic forest. Biotropica.

[34] Montade V (2019). A new modern pollen dataset describing the Brazilian Atlantic forest. The Holocene.

[35] Naafs BDA (2017). Introducing global peat-specific temperature and pH calibrations based on brGDGT bacterial lipids. Geochim. Cosmochim. Acta.

[36] Blaauw M, Christen JA (2011). Flexible paleoclimate age-depth models using an autoregressive gamma process. Bayesian Anal..

[37] Laj C, Guillou H, Kissel C (2014). Dynamics of the earth magnetic field in the 10–75 kyr period comprising the Laschamp and Mono Lake excursions: New results from the French Chaîne des Puys in a global perspective. Earth Planet. Sci. Lett..

[38] Lisiecki LE, Raymo ME (2005). A Pliocene-Pleistocene stack of 57 globally distributed benthic δ ^18^ O records. Paleoceanography.

[39] Santos TP (2017). Prolonged warming of the Brazil Current precedes deglaciations. Earth Planet. Sci. Lett..

[40] Kohfeld KE (2013). Southern hemisphere westerly wind changes during the Last Glacial Maximum: paleo-data synthesis. Quat. Sci. Rev..

[41] Cárdenas ML, Wilson OJ, Schorn LA, Mayle FE, Iriarte J (2019). A quantitative study of modern pollen–vegetation relationships in southern Brazil’s Araucaria forest. Rev. Palaeobot. Palynol..

[42] Cruz FW, Burns SJ, Karmann I, Sharp WD, Vuille M (2006). Reconstruction of regional atmospheric circulation features during the late Pleistocene in subtropical Brazil from oxygen isotope composition of speleothems. Earth Planet. Sci. Lett..

[43] Burns SJ, Welsh LK, Scroxton N, Cheng H, Edwards RL (2019). Millennial and orbital scale variability of the South American monsoon during the penultimate glacial period. Sci. Rep..

[44] Toggweiler JR, Russell JL, Carson SR (2006). Midlatitude westerlies, atmospheric CO_2_, and climate change during the ice ages: westerlies and CO_2_ during the ice ages. Paleoceanography.

[45] de Boer B, Lourens LJ, van de Wal RSW (2014). Persistent 400,000-year variability of Antarctic ice volume and the carbon cycle is revealed throughout the Plio-Pleistocene. Nat. Commun..

[46] Gu F (2017). Long-term vegetation, climate and ocean dynamics inferred from a 73,500 years old marine sediment core (GeoB2107-3) off southern Brazil. Quat. Sci. Rev..

[47] Bosmans JHC, Hilgen FJ, Tuenter E, Lourens LJ (2015). Obliquity forcing of low-latitude climate. Clim. Past.

[48] Yin Q, Berger A (2015). Interglacial analogues of the Holocene and its natural near future. Quat. Sci. Rev..

[49] Denton GH (2010). The last glacial termination. Science.

[50] Menviel L (2018). Southern hemisphere westerlies as a driver of the early deglacial atmospheric CO_2_ rise. Nat. Commun..

[51] Hirata FE, Grimm AM (2017). The role of synoptic and intraseasonal anomalies on the life cycle of rainfall extremes over South America: non-summer conditions. Clim. Dyn..

[52] Marengo J, Cornejo A, Satyamurty P, Nobre C (1997). Cold surges in tropical and extratropical South America: The strong event in June 1994. Mon. Wea. Rev.

[53] Grimm AM (2011). Interannual climate variability in South America: impacts on seasonal precipitation, extreme events, and possible effects of climate change. Stoch. Environ. Res. Risk Assess..

[54] Reimer PJ (2013). IntCal13 and Marine13 radiocarbon age calibration curves 0–50,000 years cal BP. Radiocarbon.

[55] Hua Q, Barbetti M, Rakowski AZ (2013). Atmospheric radiocarbon for the period 1950–2010. Radiocarbon.

[56] R Development Core Team. *R: A Language and Environment for Statistical Computing*. (R Foundation for Statistical Computing, Vienna, Austria, 2019).

[57] Aitken, M. J. *An introduction to optical dating: the dating of Quaternary sediments by the use of photon-stimulated luminescence*. (Oxford University Press, 1998).

[58] Murray AS, Wintle AG (2003). The single aliquot regenerative dose protocol: potential for improvements in reliability. Radiat. Meas..

[59] Jain M, Duller GAT, Wintle AG (2007). Dose response, thermal stability and optical bleaching of the 310 °C isothermal TL signal in quartz. Radiat. Meas..

[60] Buylaert J-P (2012). A robust feldspar luminescence dating method for middle and late Pleistocene sediments: Feldspar luminescence dating of middle and late Pleistocene sediments. Boreas.

[61] Galbraith, R. F., Roberts, R. G., Laslett, G. M., Yoshida, H. & Olley, J. M. Optical dating of single and multiple grains of quartz from minmium rock shelter, northern Australia: part i, experimental design and statistical models. *Archaeometry* **41**, 339–364 (1999).

[62] Guérin G, Mercier N, Adamiec G (2011). Dose-rate conversion factors: update. Ancient TL.

[63] Prescott J, Stephan L (1982). The contribution of cosmic radiation to the environmental dose for thermoluminescent dating, latitude, altitude and depth dependences. PACT.

[64] Channell JET, Xuan C, Hodell DA (2009). Stacking paleointensity and oxygen isotope data for the last 1.5 Myr (PISO-1500). Earth Planet. Sci. Lett..

[65] Simon Q (2016). Authigenic ^10^ Be/^9^ Be ratio signatures of the cosmogenic nuclide production linked to geomagnetic dipole moment variation since the Brunhes/Matuyama boundary. J. Geophys. Res. Solid Earth.

[66] Simon Q, St‐Onge G, Hillaire‐Marcel C (2012). Late Quaternary chronostratigraphic framework of deep Baffin Bay glaciomarine sediments from high‐resolution paleomagnetic data. Geochem. Geophys. Geosystems.

[67] Thouveny N, Creer KM, Blunk I (1990). Extension of the Lac du Bouchet palaeomagnetic record over the last 120,000 years. Earth Planet. Sci. Lett..

[68] Croudace IW, Rindby A, Rothwell RG (2006). ITRAX: description and evaluation of a new multi-function X-ray core scanner. Geol. Soc. Lond. Spec. Publ..

[69] Chawchai S, Kylander ME, Chabangborn A, Löwemark L, Wohlfarth B (2016). Testing commonly used X-ray fluorescence core scanning-based proxies for organic-rich lake sediments and peat. Boreas.

[70] Hopmans EC, Schouten S, Sinninghe Damsté JS (2016). The effect of improved chromatography on GDGT-based palaeoproxies. Org. Geochem..

[71] Davtian N, Bard E, Ménot G, Fagault Y (2018). The importance of mass accuracy in selected ion monitoring analysis of branched and isoprenoid tetraethers. Org. Geochem..

[72] Davtian N, Ménot G, Fagault Y, Bard E (2019). Western mediterranean sea paleothermometry over the last glacial cycle based on the novel RI-OH index. Paleoceanogr. Paleoclimatology.

[73] Sanchi L, Ménot G, Bard E (2013). An automated purification method for archaeal and bacterial tetraethers in soils and sediments. Org. Geochem..

[74] De Jonge C (2014). Occurrence and abundance of 6-methyl branched glycerol dialkyl glycerol tetraethers in soils: Implications for palaeoclimate reconstruction. Geochim. Cosmochim. Acta.

[75] Russell JM, Hopmans EC, Loomis SE, Liang J, Sinninghe Damsté JS (2018). Distributions of 5- and 6-methyl branched glycerol dialkyl glycerol tetraethers (brGDGTs) in East African lake sediment: Effects of temperature, pH, and new lacustrine paleotemperature calibrations. Org. Geochem..

[76] Hopmans EC (2004). A novel proxy for terrestrial organic matter in sediments based on branched and isoprenoid tetraether lipids. Earth Planet. Sci. Lett..

[77] Weijers JWH, Schouten S, Spaargaren OC, Sinninghe Damsté JS (2006). Occurrence and distribution of tetraether membrane lipids in soils: Implications for the use of the TEX86 proxy and the BIT index. Org. Geochem..

[78] De Jonge C (2013). Identification of novel penta- and hexamethylated branched glycerol dialkyl glycerol tetraethers in peat using HPLC–MS2, GC–MS and GC–SMB-MS. Org. Geochem..

[79] Xiao W (2016). Ubiquitous production of branched glycerol dialkyl glycerol tetraethers(brGDGTs) in global marine environments: a new source indicator for brGDGTs. Biogeosciences.

[80] Martin C (2020). Early Holocene Thermal Maximum recorded by branched tetraethers and pollen in Western Europe (Massif Central, France). Quat. Sci. Rev..

[81] Stute M (1995). Cooling of tropical Brazil (5 °C) during the Last Glacial Maximum. Science.

[82] Faegri, K. & Iversen, J. *Textbook of Pollen Analysis* (John Wiley & Sons, Chichester, 1989).

[83] Bennett, K. *Psimpoll 4.27: C program for plotting pollen diagrams and analysing pollen data*. (Department of Archaeology and Palaeoecology, Queen’s University of Belfast, 2009).

[84] Daniau A-L (2013). Orbital-scale climate forcing of grassland burning in southern Africa. Proc. Natl. Acad. Sci..

[85] Adler RF (2003). The version-2 global precipitation climatology project (GPCP) monthly precipitation analysis (1979–Present). J. Hydrometeorol..

[86] Laskar J (2004). A long-term numerical solution for the insolation quantities of the Earth. Astron. Astrophys..

[87] Railsback LB, Gibbard PL, Head MJ, Voarintsoa NRG, Toucanne S (2015). An optimized scheme of lettered marine isotope substages for the last 1.0 million years, and the climatostratigraphic nature of isotope stages and substages. Quat. Sci. Rev..

[88] Henderson GM, Slowey NC (2000). Evidence from U–Th dating against Northern Hemisphere forcing of the penultimate deglaciation. Nature.

[89] Lüthi D (2008). High-resolution carbon dioxide concentration record 650,000–800,000 years before present. Nature.

