# Supplementary material for: Shut down of the South American summer monsoon during the penultimate glacial
Source: Sci Rep. 2020 Apr 15;10:6275. doi: 10.1038/s41598-020-62888-x (PMC7160121; doi:10.1038/s41598-020-62888-x)
Supplement: Supplementary file 1 — Supplementary Material. [file 41598_2020_62888_MOESM1_ESM.docx]

**Supplementary material**

**Shut down of the South American summer monsoon during the penultimate glacial**

**Authors:** Paula A. Rodríguez-Zorro^1*^, Marie-Pierre Ledru^1^, Edouard Bard^2^, Olga Aquino-Alfonso^1^, Adriana Camejo^3^, Anne-Laure Daniau^4^, Charly Favier^1^, Marta García^2^, Thays D. Mineli^5^, Frauke Rostek^2^, Fresia Ricardi-Branco^3^, André Oliveira Sawakuchi^5^, Quentin Simon^2^, Kazuyo Tachikawa^2^, Nicolas Thouveny^2^

**Affiliations:**

**1.** Institut des Sciences de l’Evolution-Montpellier (ISEM), Univ Montpellier, CNRS, IRD, EPHE, 34095 Montpellier, France

**2.** Aix Marseille Univ, CNRS, IRD, INRAE, Coll France, CEREGE, 13545, Aix-en-Provence, France

**3.** Institute of Geosciences, University of Campinas, R. Pandiá Calógeras 51, Cidade Universitária Zeferino Vaz, 13081-970 Campinas, Brazil

**4.** Environnements et paléoenvironnements océaniques et continentaux (EPOC), UMR 5805 CNRS, University of Bordeaux, 33615 Pessac, France

**5.** Institute of Geosciences, University of São Paulo, São Paulo, Brazil

* Corresponding author: [paularsat@gmail.com](mailto:paularsat@gmail.com)

**
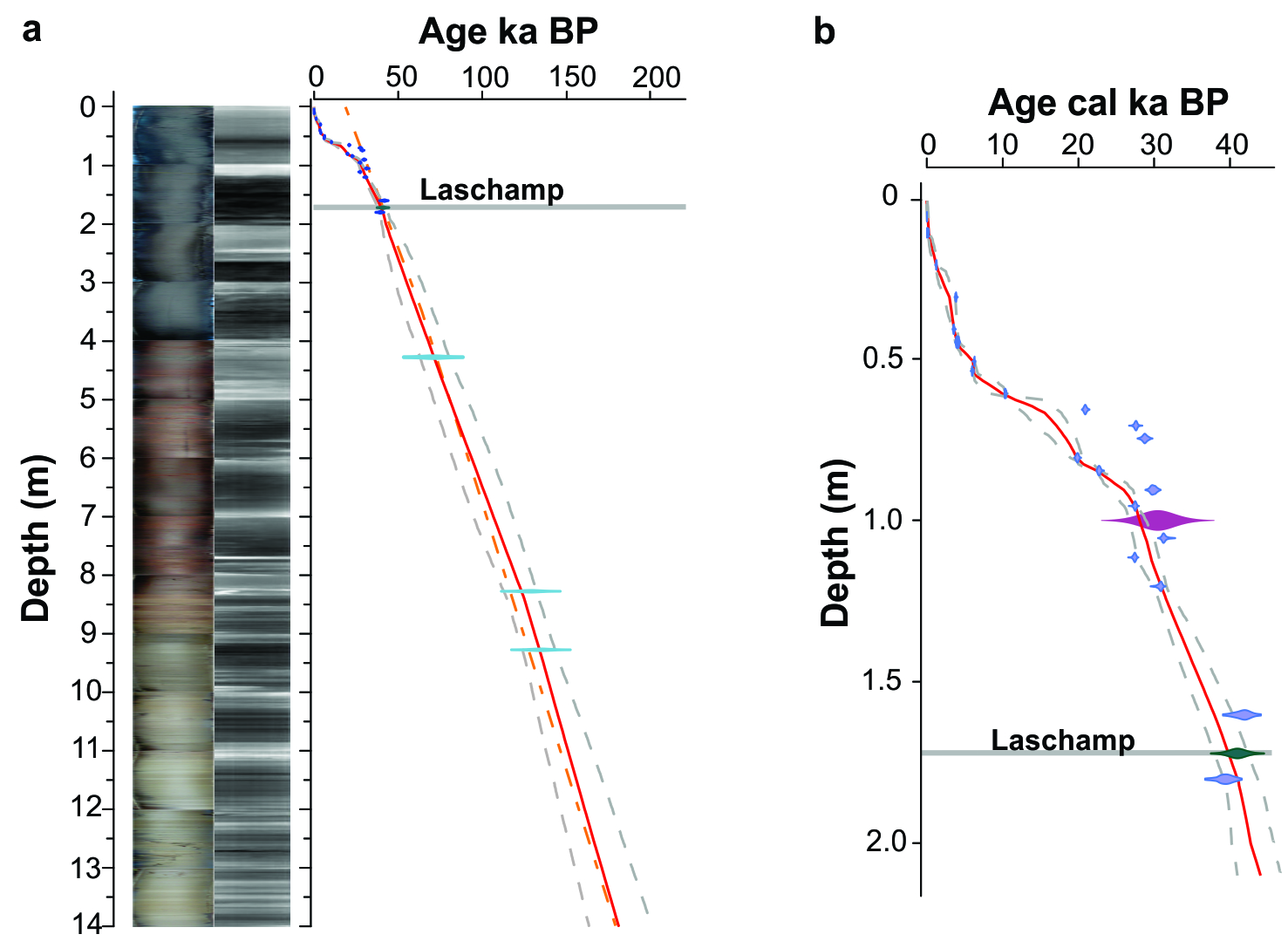
**

**Fig S1. CO14 age-depth model.** **a.** lithology and X-ray of the CO14 record, the Bayesian^1^ age-depth model; the MAAT tie points are in light blue (see supplementary Fig. 2).**b.** Details of the Bayesian age-depth model for the radiocarbon ages (dark blue) Laschamp^2^ geomagnetic reversal (green) tie point. The OSL date (purple; not included in the calculations) is showing the agreement with the obtained model (see Methods section). Grey dashed lines represent the 95% confidence intervals from the Bayesian age-depth model CO14. The red curve shows the single best model based on the median age for each depth from CO14. The orange dashed line in panel a. compares the model obtained by fine-tuning of the RPI curve to two independent paleointensity reference curves^3,4^ (see Supplementary Fig. 3).

**
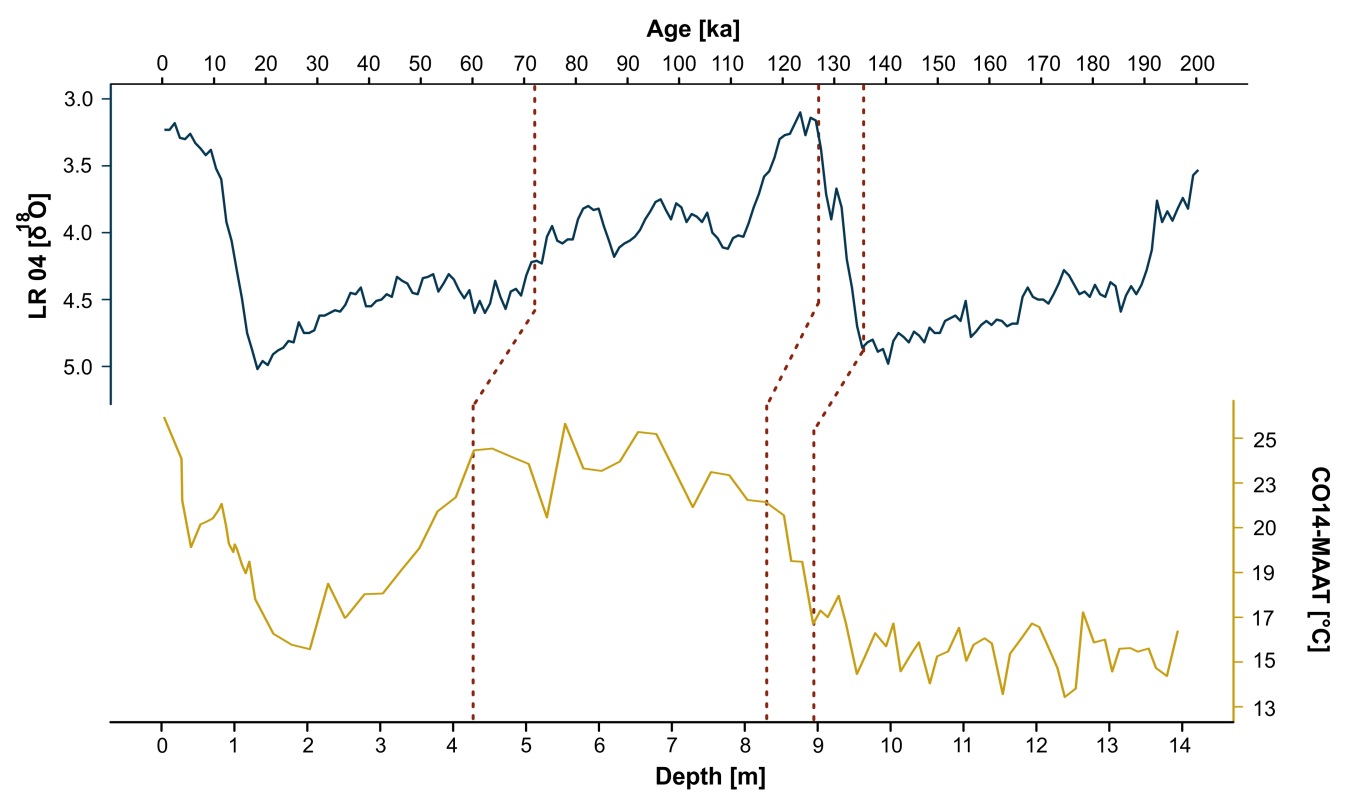
**

**Fig. S2. Selected LR04 δ^18^O stack^5^ tie points compared to the MAAT from CO14.** Red dashed lines indicate the selected tie points between both curves. Bottom part of the CO14-MAAT shows no clear contrasting change in temperature, indicating that the record is not older than 191 ka.

**
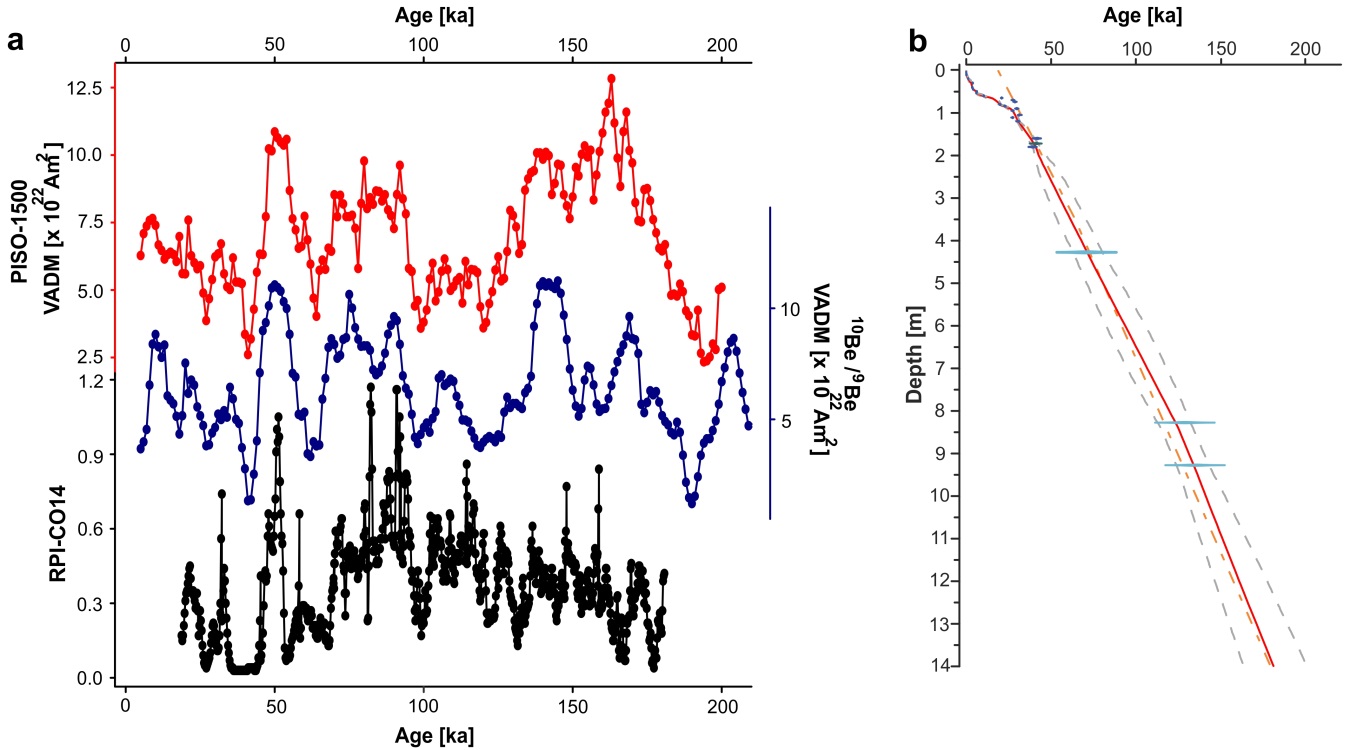
**

**Fig S3. Magnetostratigraphy tuning and Bayesian age-depth model from CO14,**

**a.** RPI tuning curves. The calibrated RPI PISO-1500^3^ in Virtual Axial Dipole Moment (VADM) units (in red), the calibrated ^10^ Be / ^9^ Be ratio^4^ (in blue), RPI index from Colônia (CO14 record) (in black). **b.** Grey dashed lines show the 95% confidence intervals from the Bayesian age-depth model CO14. The red curve shows the single best model based on the median age for each depth from CO14. The orange dashed line represents the model obtained by fine-tuning the RPI curve to two independent paleointensity reference curves^3,4^ used as a posteriori validation. Radiocarbon dates are in dark blue, Laschamp^2^ tie point in dark green and temperature tie points are in light blue.

**
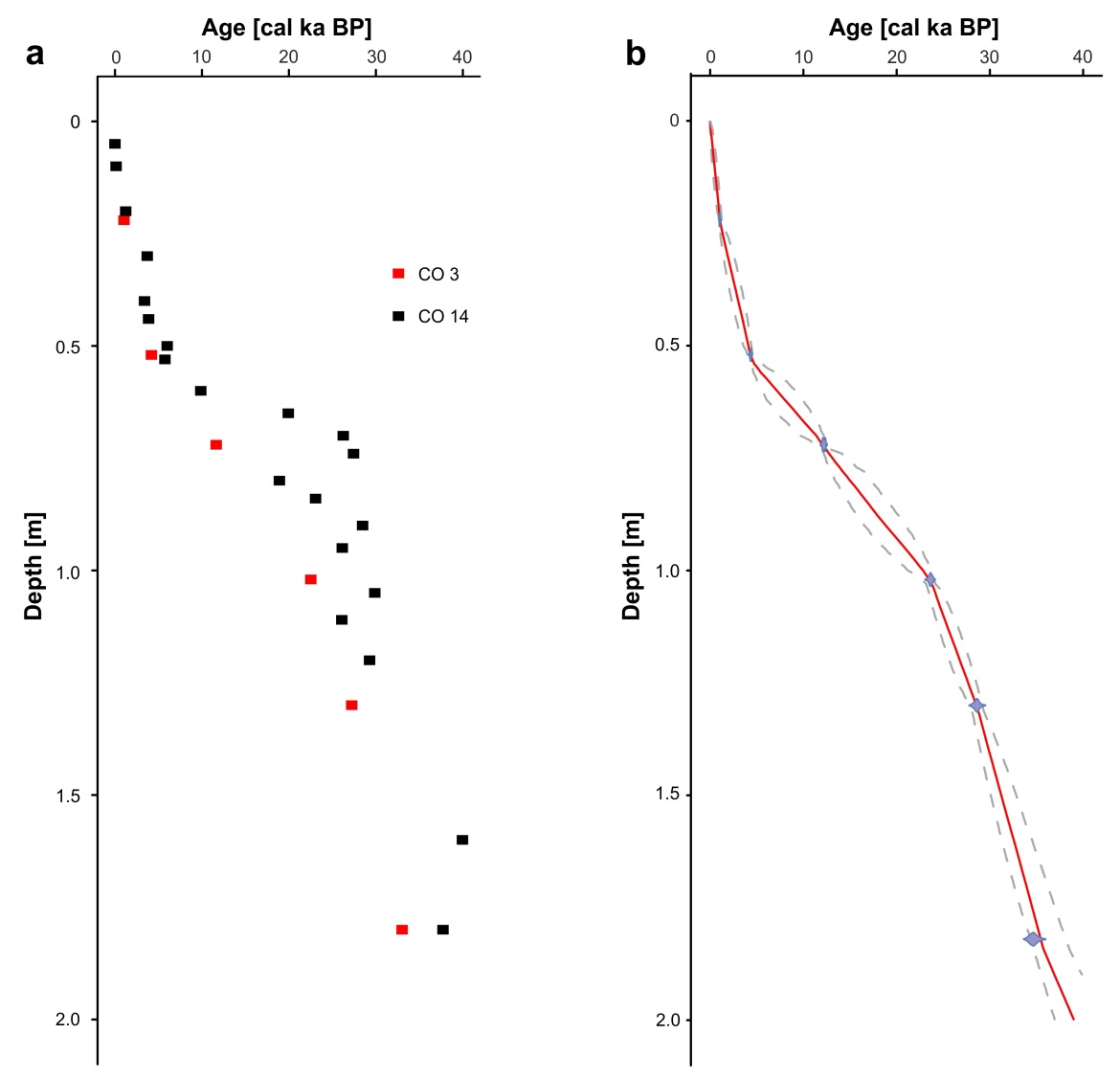
**

**Fig. S4.CO3 and CO14 alignment parameters a.**CO3 and CO14 ^14^C calibrated ages, showing the similar ages in the parallel records. **b.**Bayesian age-depth model from CO3**.** Grey dashed lines represent the 95% confidence intervals from the Bayesian age-depth model^2^ CO3. The red curve shows the single best model based on the median age for each depth from CO3.

**
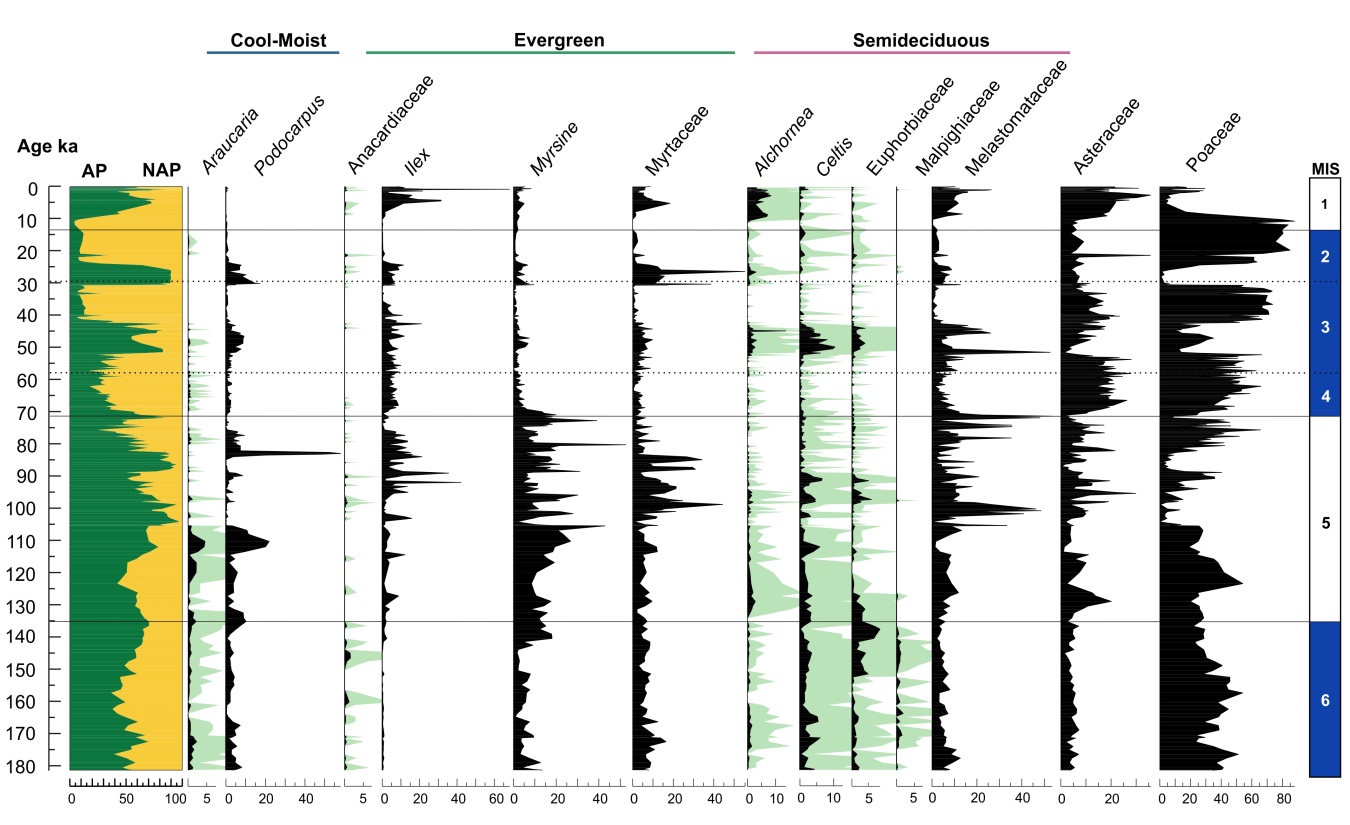
**

**Fig. S5. Pollen percentage diagram of selected taxa in the Colônia record**. From left to right, Ages ka; Summary diagram of arboreal pollen (AP) and non-arboreal pollen (NAP) from Colônia. Selected cool moist taxa indicators (blue line); the most relevant evergreen forest taxa indicators (green line); the most relevant semi-deciduous taxa indicators (pink line); most relevant open areas taxa indicators. Marine Isotopic divisions; glacial periods are highlighted in blue. Green shading in taxa with less than 10% is an exaggeration by 10. The marine isotopic stage boundaries ages are based on the LR04 δ^18^O record^5,6^. The boundary between MIS 5 and 6 is based on Henderson and Slowey^7^.

**
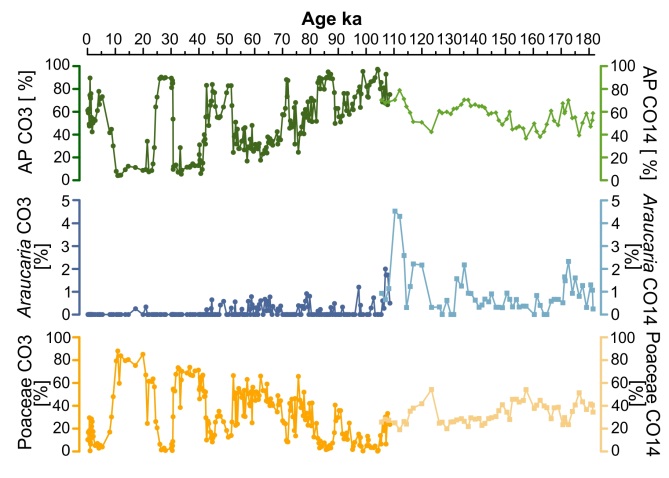
**

**Fig S6. Selected pollen percentage curves from CO3 and CO14** highlighting the matching sections in the two pollen datasets. AP (Arboreal Pollen %)

**
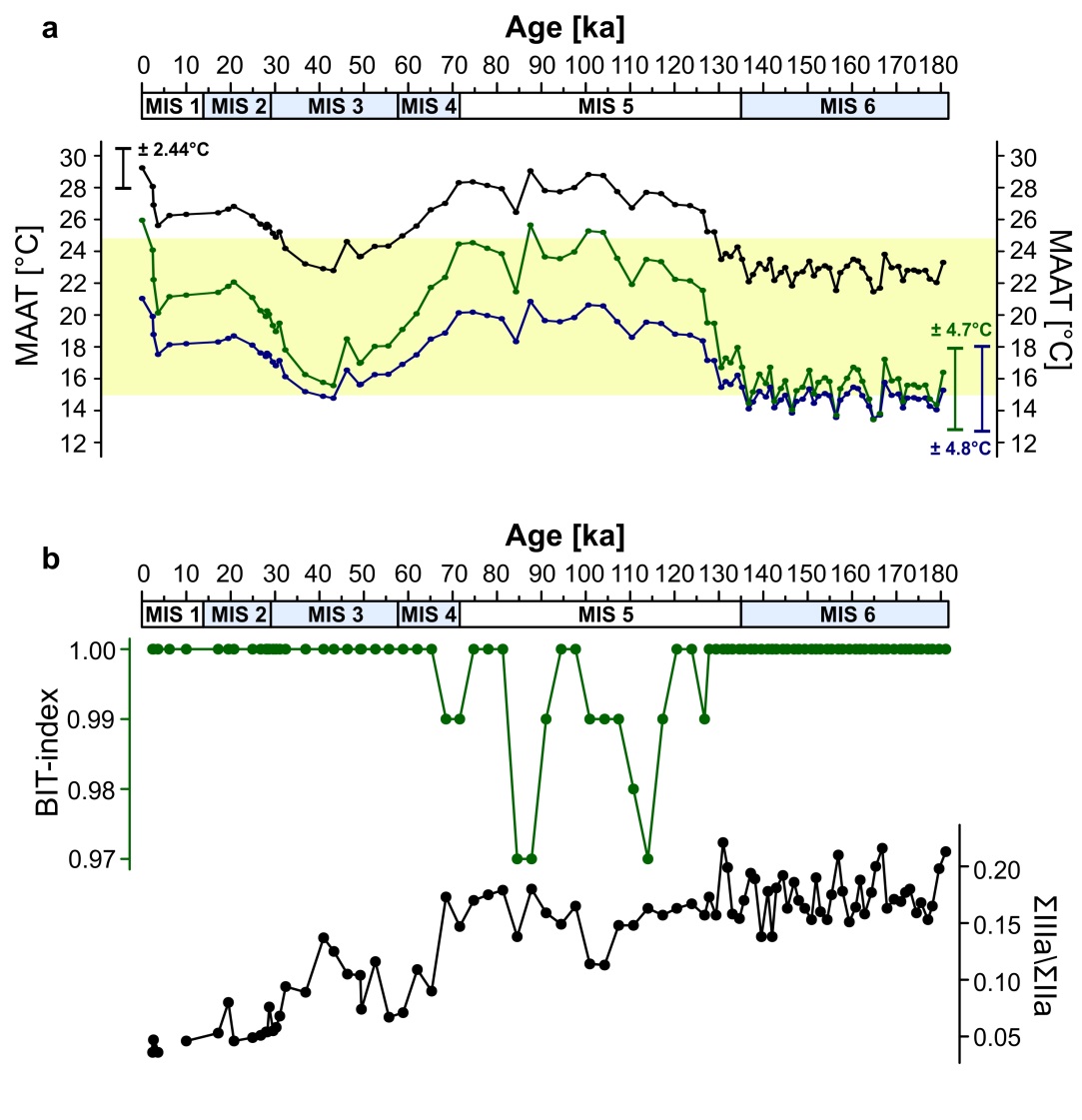
**

**Fig.S7. MAAT calibrated curves based on the MBT’5ME index and Origin of the brGDGTs from CO14. a.** Lake calibration (Russell et al.^8^; black curve; RMSE ≈ ± 2.44), peat calibration of Naafs et al.^9^ (green curve; RMSE ≈ ± 4.7 ) and soil calibration (De Jonge et al^10^ ; blue curve; RMSE ≈ ± 4.8). Beige band highlights the current seasonal temperature variation in the region. The overall shapes and amplitudes are similar. The lake (black) calibration leads to too high temperatures, notably the core top would be much warmer than the modern temperature, which rules out a dominant in situ aquatic production within the lake. **b.** In green, branched and Isoprenoid Tetraether index (BIT) and in black, ∑IIIa/∑IIa ratio of brGDGTs from CO14 record. High BIT (>0.97) and low ∑IIIa/∑IIa (<0.2) values indicate the terrestrial origin for brGDGTs.

**
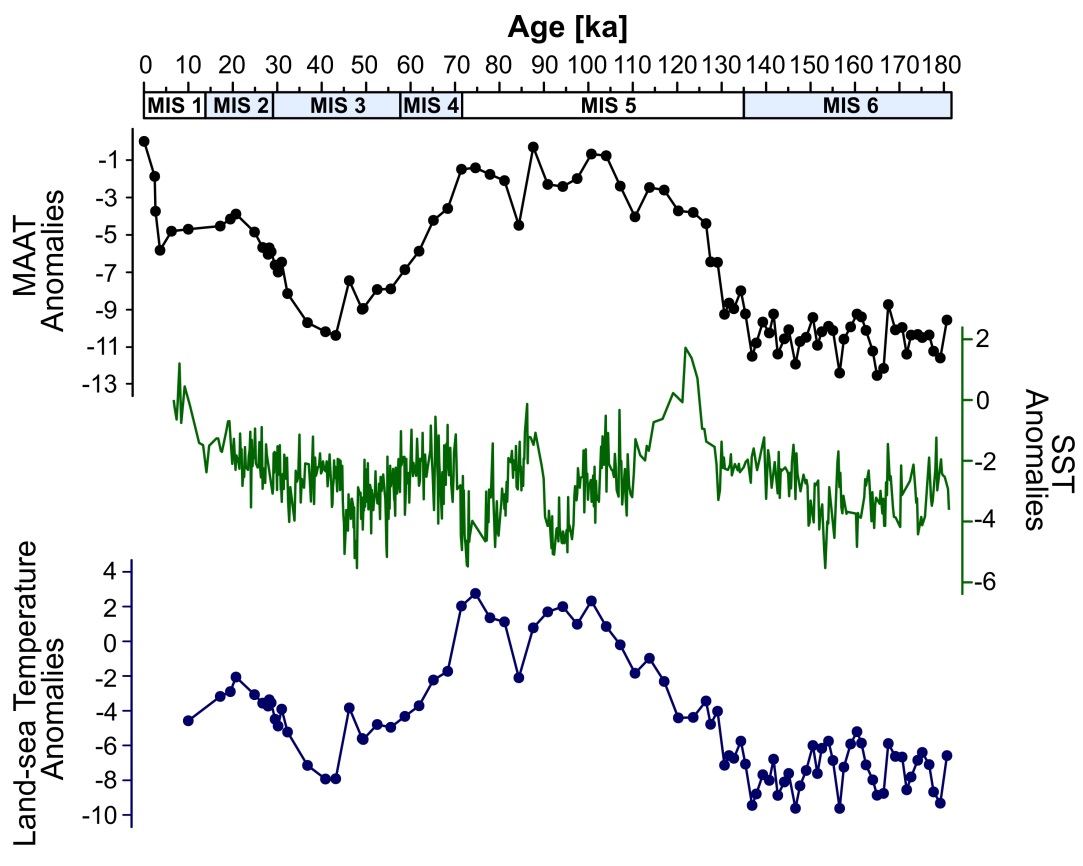
**

**Fig.S8. Land-sea temperature anomalies at latitud 23-25° S. a.** MAAT from CO14 record.**b.** GL-1090 (Santos et al.^11^): *Globigerinoides ruber* Mg/Ca sea surface temperature (SST) **c.** Land-sea temperature curve between the MAAT from Colônia and the SST from the GL-1090 (Santos et al.^11^)

**
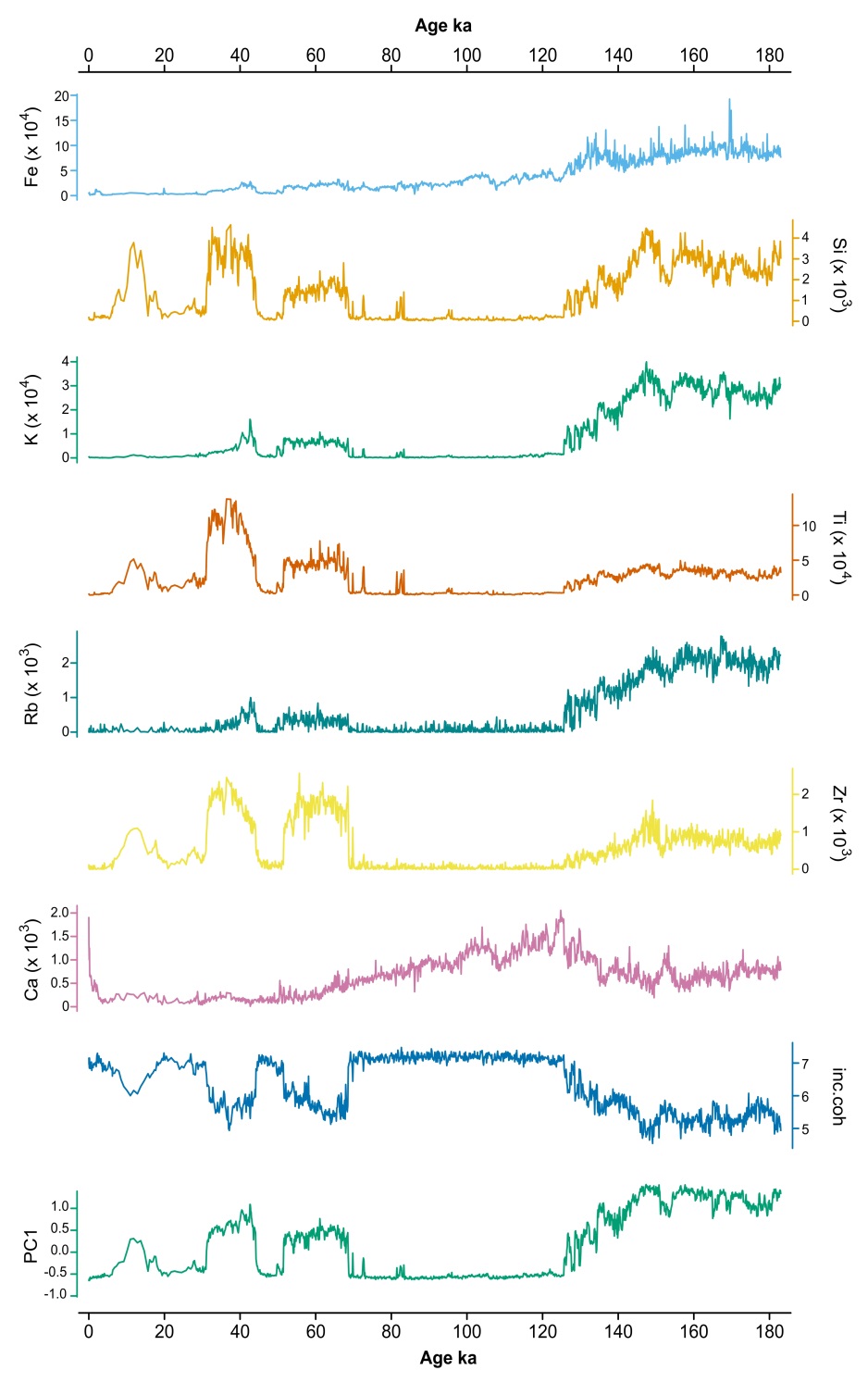
**

**Fig S9. Intensity of CO14 XRF elements.**

**
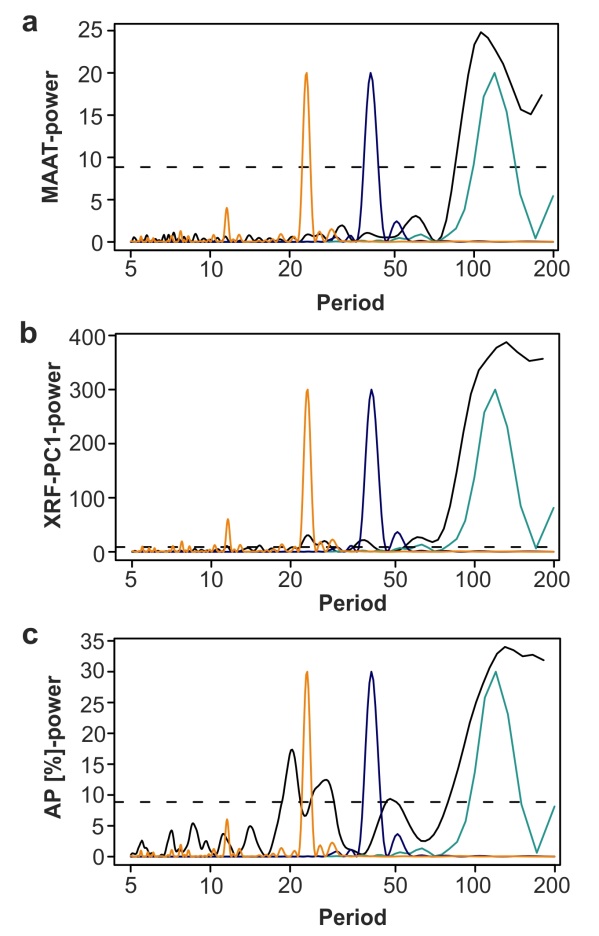
**

**Fig. S10. Least squares spectral analysis (LSSA) from selected Colônia datasets** compared with eccentricity, obliquity and precession signals^12^ (turquoise, blue and orange curves respectively) using the lomb^13^ package in R^14^ **a**. MAAT. **b.**XRF PC1 **c.** Arboreal Pollen (AP). The black line represents the LSSA of each proxy. The black dashed line is the threshold between the signal and noise.

**Description of the sediments.** The sediments from the CO14 record are characterised by a silty-clayey lacustrine section between 14 to 850 cm in depth and an organic peat section towards the top of the record. The lacustrine section is characterised by clear light and dark laminations with larger amounts of mica plates in coarser grained bands. These changes in detrital input reflect the variability of the intensity of XRF elements (e.g. PC1, Si, Ti, K, inc/coh) that differentiate peatland from lake sediments in the CO14 record (Supplementary Fig.S9). Coarser sediments and large amounts of mica in the lacustrine section reveal the influence of one or more of the streams flowing from the hills along the rim, since geologically the rim of the crater is dominated by mica schist and quartzite^15^. Conversely, in the peat section, the high detrital input phases appear to be linked with the opening of the landscape and erosion in the coring site (Fig.1). The increase in the supply of sediments is linked to eroded sediments transported during sporadic wet events.

**Table S1**. AMS radiocarbon dates from CO14

| **Sample ID** | **Batch** | **Depth CO14 [m]** | **pMC** | **δ^13^C [‰]** | **^14^C Age [ka BP]** | **Min age [ka cal BP]** | **Max age [ka cal BP]** | **Age interval probability [%]** |
| --- | --- | --- | --- | --- | --- | --- | --- | --- |
| SacA41593 | 1 | 0.05 | 103.07 ± 0.28 | -29.56 | -243 ± 22 | 1957 cal AD* | | 95 |
| SacA44991 | 2 | 0.1 | 98.53 ± 0.24 | -32.2 | 120 ± 30 | 0.11 | 0.49 | 15.2 |
|  |  |  |  |  |  | 0.51 | 0.149 | 47.4 |
|  |  |  |  |  |  | 0.187 | 0.270 | 32.3 |
| SacA41594 | 1 | 0.2 | 84.39 ± 0.27 | -30.58 | 1365 ± 30 | 1.19 | 1.2 | 1 |
|  |  |  |  |  |  | 1.26 | 1.33 | 93.9 |
| SacA44992 | 2 | 0.3 | 64.01 ± 0.23 | -24.1 | 3585 ± 30 | 3.83 | 3.98 | 95 |
| SacA41595 | 1 | 0.4 | 65.92 ± 0.31 | -18.18 | 3350 ± 40 | 3.48 | 3.65 | 86.4 |
|  |  |  |  |  |  | 3.66 | 3.69 | 8.6 |
| SacA44993 | 2 | 0.44 | 62.84 ± 0.20 | -25.5 | 3730 ± 30 | 3.98 | 4.15 | 95 |
| SacA44994 | 2 | 0.5 | 50.35 ± 0.20 | -26.2 | 5510 ± 30 | 6.22 | 6.23 | 0.9 |
|  |  |  |  |  |  | 6.28 | 6.35 | 78.3 |
|  |  |  |  |  |  | 6.36 | 6.40 | 15.7 |
| SacA44995 | 2 | 0.53 | 51.89 ± 0.20 | -27.6 | 5270 ± 30 | 5.94 | 5.98 | 13.3 |
|  |  |  |  |  |  | 5.98 | 6.12 | 65.7 |
|  |  |  |  |  |  | 6.15 | 6.18 | 15.8 |
| SacA41596 | 1 | 0.6 | 31.70 ± 0.18 | -21.16 | 9225 ± 45 | 10.26 | 10.51 | 95 |
| SacA44996 | 2 | 0.65 | 11.5 ± 0.15 | -26.6 | 17370 ± 100 | 20.67 | 21.28 | 95 |
| SacA44997 | 2 | 0.7 | 5.40 ± 0.16 | -26.5 | 23440 ± 230 | 27.29 | 27.97 | 95 |
| SacA44998 | 2 | 0.74 | 4.57 ± 0.15 | -10.7 | 24780 ± 260 | 28.31 | 29.44 | 95 |
| SacA41597 | 1 | 0.8 | 12.81 ± 0.16 | -26.85 | 16510 ± 100 | 19.62 | 20.17 | 95 |
| SacA44999 | 2 | 0.84 | 9.49 ± 0.13 | -25.3 | 18920 ± 110 | 22.49 | 23.06 | 95 |
| SacA45000 | 2 | 0.9 | 4.07 ± 0.12 | -27.3 | 25720 ± 240 | 29.34 | 30.60 | 95 |
| SacA45001 | 2 | 0.95 | 5.51 ± 0.12 | -22.2 | 23270 ± 180 | 27.24 | 27.78 | 95 |
| SacA41598 | 1 | 1.05 | 3.29 ± 0.14 | -28.25 | 27420 ± 340 | 30.9 | 32.0 | 95 |
| SacA45002 | 2 | 1.11 | 5.58 ± 0.12 | -22.3 | 23190 ± 180 | 27.17 | 27.74 | 95 |
| SacA41599 | 1 | 1.2 | 3.61 ± 0.14 | -28.53 | 26690 ± 310 | 30.4 | 31.3 | 95 |
| SacA41600° | 1 | 1.4 | < 0.445 | -25.92 | > 43500 | > 43.5 |  |  |
| SacA41601 | 1 | 1.6 | 0.92 ± 0.13 | -27.32 | 37600 ± 1200 | 40.1 | 44.0 | 95 |
| SacA41602 | 1 | 1.8 | 1.29 ± 0.13 | -27.13 | 34960 ± 840 | 38.0 | 41.4 | 95 |

*Calibrated using the postbomb curve for the Southern Hemisphere^12^

° Discarded age for age model

**Table S2**. Luminescence dates in sandy intervals obtained from sediment core CO14.

| **Sample ID** | **Depth CO14 [m]** | **Method** | **Mineral** | **Number of Aliquots** | **Dose[Gy]** | **Dose rate [Gy/ka]** | **Age [ka]** |
| --- | --- | --- | --- | --- | --- | --- | --- |
| L0247 | 1 | OSL | Quartz | 6 | 29.98 ± 3.16 | 0.95 ± 0.08 | 31.4 ± 4.3 |
| L0250* | 11 | ITL | Quartz | 5 | 669.35 ± 141.26 | 3.65 ± 0.29 | 183.3 ± 41.3 |
|  |  | IR50 | K-feldspar | 4 | 631.95 ± 95.75 | 4.08 ± 0.29 | 154.9 ± 25. |

*discarded ages

**Table S3**. Selected tie points from reconstructed MAAT °C curve and paleomagnetic event.

| **Depth CO14 (m)** | **Calendar age (ka BP)** | **Periods** | **Calibration data** |
| --- | --- | --- | --- |
| 8.275 | 129 ± 5 | Onset MIS 5e | Lisiecki and Raymo (2005) |
| 8.925 | 135 ± 5 | MIS 6/5 | Lisiecki and Raymo (2005); Henderson and Slowey (2000) |
| 4.275 | 71 ± 5 | MIS 5/4 | Lisiecki and Raymo (2005) |
| 1.72 | 41 ± 1 | Laschamp | Laj et al., 2014 |

**Table 4** AMS radiocarbon dates from CO3^16^

| **Sample ID** | **Depth CO3 (m)** | **δ^13^C [‰]** | **14C Age**  **[yr BP]** | **Min age**  **[ka cal**  **BP]** | **Max age**  **[ka cal**  **BP]** | **Age interval**  **Probability**  **[%]** |
| --- | --- | --- | --- | --- | --- | --- |
| LY500 | 0.22 | -28.47 | 1170 ± 35 | 0.982 | 1.036 | 17.4 |
|  |  |  |  | 1.043 | 1.179 | 77.5 |
| LY501 | 0.52 | -27.6 | 3955 ± 30 | 4.29 | 4.33 | 11 |
|  |  |  |  | 4.35 | 4.45 | 57.4 |
|  |  |  |  | 4.46 | 4.52 | 26.6 |
| LY502 | 0.72 | -23.11 | 10400 ± 60 | 12.05 | 12.35 | 88.3 |
|  |  |  |  | 12.46 | 12.53 | 6.6 |
| LY503 | 1.02 | -23.48 | 19670 ± 110 | 23.40 | 24.00 | 95 |
| LY504 | 1.30 | -28.72 | 24615 ± 225 | 28.14 | 29.17 | 95 |
| LY505 | 1.82 | -24.27 | 30750 ± 400 | 34.01 | 35.50 | 95 |

**References**

1. Blaauw, M. & Christen, J. A. Flexible paleoclimate age-depth models using an autoregressive gamma process. *Bayesian Anal.* **6,** 457–474 (2011).

2. Laj, C., Guillou, H. & Kissel, C. Dynamics of the earth magnetic field in the 10–75 kyr period comprising the Laschamp and Mono Lake excursions: New results from the French Chaîne des Puys in a global perspective. *Earth Planet. Sci. Lett*. **387,** 184–197 (2014).

3. Channell, J. E. T., Xuan, C. & Hodell, D. A. Stacking paleointensity and oxygen isotope data for the last 1.5 Myr (PISO-1500). *Earth Planet. Sci. Lett*. **283,** 14–23 (2009).

4. Simon, Q. *et al.* Authigenic ^10^Be/ ^9^Be ratio signatures of the cosmogenic nuclide production linked to geomagnetic dipole moment variation since the Brunhes/Matuyama boundary. *J. Geophys. Res. Solid Earth* **121,** 7716–7741 (2016).

5. Lisiecki, L. E. & Raymo, M. E. A Pliocene-Pleistocene stack of 57 globally distributed benthic δ 18 O records. *Paleoceanography* **20,** 1–17 (2005).

6. Railsback, L. B., Gibbard, P. L., Head, M. J., Voarintsoa, N. R. G. & Toucanne, S. An optimized scheme of lettered marine isotope substages for the last 1.0 million years, and the climatostratigraphic nature of isotope stages and substages. *Quat. Sci. Rev.* **111,** 94–106 (2015).

7. Henderson, G. M. & Slowey, N. C. Evidence from U–Th dating against Northern Hemisphere forcing of the penultimate deglaciation. *Nature* **404,** 61–66 (2000).

8. Russell, J. M., Hopmans, E. C., Loomis, S. E., Liang, J. & Sinninghe Damsté, J. S. Distributions of 5- and 6-methyl branched glycerol dialkyl glycerol tetraethers (brGDGTs) in East African lake sediment: Effects of temperature, pH, and new lacustrine paleotemperature calibrations. *Org. Geochem.* **117,** 56–69 (2018).

9. Naafs, B. D. A. *et al.* Introducing global peat-specific temperature and pH calibrations based on brGDGT bacterial lipids. *Geochim. Cosmochim. Acta* **208,** 285–301 (2017).

10. De Jonge, C. *et al.* Occurrence and abundance of 6-methyl branched glycerol dialkyl glycerol tetraethers in soils: Implications for palaeoclimate reconstruction. *Geochim. Cosmochim. Acta* **141,** 97–112 (2014).

11. Santos, T. P. *et al.* Prolonged warming of the Brazil Current precedes deglaciations. *Earth Planet. Sci. Lett.* **463,** 1–12 (2017).

12. Laskar, J. *et al.* A long-term numerical solution for the insolation quantities of the Earth. *Astron. Astrophys.* **428,** 261–285 (2004).

13. Ruf, T. The Lomb-Scargle Periodogram in Biological Rhythm Research: Analysis of Incomplete and Unequally Spaced Time-Series. *Biol. Rhythm Res.* **30,** 178–201 (1999).

14. R Development Core Team. *R: A Language and Environment for Statistical Computing*. (R Foundation for Statistical Computing, Vienna, Austria, 2019).

15. Riccomini, C. *et al.* The Colônia structure, São Paulo, Brazil. *Meteorit. Planet. Sci.* **46,** 1630–1639 (2011).

16. Ledru, M.-P., Mourguiart, P. & Riccomini, C. Related changes in biodiversity, insolation and climate in the Atlantic rainforest since the last interglacial. *Palaeogeogr. Palaeoclimatol. Palaeoecol.* **271,** 140–152 (2009).
